# Pre-association enables visible-light induced proton-coupled electron-transfer in a titanium-functionalized polyoxotungstate

**DOI:** 10.1039/d6sc04575h

**Published:** 2026-07-13

**Authors:** Paul Kadereit, Kristin Sellmann, Dayana M. Galeas, Sayan Kangsa Banik, Martin Diefenbach, Dirk Schwarzer, Vera Krewald, Carsten Streb

**Affiliations:** a Department of Chemistry, Johannes Gutenberg University Mainz Duesbergweg 10-14 55128 Mainz Germany carsten.streb@uni-mainz.de; b Department of Chemistry, TU Darmstadt Peter-Grünberg-Str. 4 64287 Darmstadt Germany vera.krewald@tu-darmstadt.de; c Department of Dynamics at Surfaces, Max Planck Institute for Multidisciplinary Sciences Am Fassberg 11 37077 Göttingen Germany dschwar@mpinat.mpg.de

## Abstract

Proton-coupled electron transfer (PCET) is central to many chemical transformations and catalytic processes. Heterometal-doped polyoxometalates (POMs) offer powerful model systems for bulk metal oxides to explore this fundamental reactivity. In this work we study the mechanism of the selective photochemical oxidation of benzyl alcohol to benzaldehyde facilitated by the titanium-doped Keggin polyoxotungstate [(TiOH)PW_11_O_39_]^4−^{TiOH}. In aqueous solution, reaction of this cluster with benzyl alcohol results in formation of an unusual, visible-light photoactive intermediate, [(TiOBn)PW_11_O_39_]^4−^{TiOBn}, which is capable of undergoing photoinduced PCET, resulting in the oxidation of benzyl alcohol to benzaldehyde, and simultaneous reduction/protonation of the POM. Experimental and theoretical studies are combined to provide comprehensive insights into the underlying mechanisms, to explain the unusual properties of the POM-alkoxide intermediate, and to probe the chemical and photophysical properties of the species involved in the photo-oxidation process. Non-aqueous Pourbaix analysis is used to provide further understanding of the PCET reactivity of the POM. This work provides unique insights into the design of noble metal-free, visible light-photoactive molecular metal oxides for selective organic photooxidation reactions in water.

## Abstract 2

Combined experimental and theoretical studies shed light on the proton-coupled electron transfer mechanism of a polyoxometalate-based alcohol oxidation in water, highlighting the importance of substrate pre-coordination to the reactive site.

## Graphical abstract

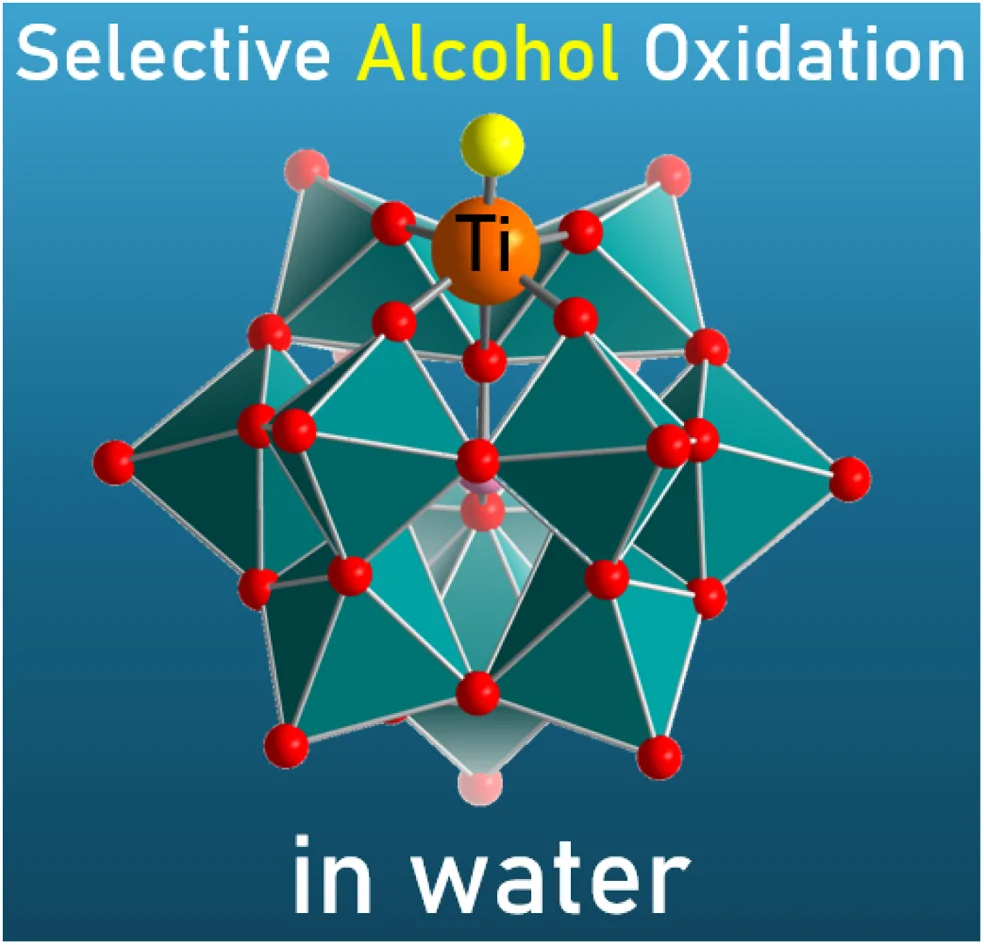

## Introduction

C–H bond activation is a key chemical reaction and impacts areas from metabolic reactions in living systems to large-scale industrial processes.^1^ The activation of low-polarity C–H bonds requires significant energy input, since most C_sp3_–H bonds have bond dissociation energies (BDEs) in the range of *ca.* 390–440 kJ mol^−1^.^2,3^ Therefore, selective C–H activation to form one specific target product is still a major challenge, as selectivity is difficult to control.^1^ Over recent years, heterogeneous (photo-) reducible transition metal oxides, *e.g.* ZnO, TiO_2_ or WO_3_ have emerged as earth-abundant materials for selective C–H activations.^4–6^ This is due to their ability to facilitate proton-coupled electron transfer (PCET) at their reactive surfaces.^5,7,8^ Pioneering studies have shown that these species can facilitate a range of C–H activations using PCET as a key mechanism.^5,7,9,10^ In a seminal recent study, Han, Niu, Tang and coworkers demonstrated the selective methane-to-formaldehyde oxidation using a WO_3_ photocatalyst.^11^ The group assigned this reactivity to a facet-selective competitive pre-association of methane, resulting in high selectivity rather than leading to unselective radical pathways. In a related study, Zhao and coworkers facilitated selective photooxidation of amines using TiO_2_ as light-absorber and reaction site. The selectivity observed was assigned to pre-coordination of the substrate to the TiO_2_ surface, resulting in activation of the α-CH group.^12^ These initial studies suggest that thermodynamic control and understanding of the PCET mechanism allow fine-tuning of reaction kinetics and thereby control over product selectivity. However, to-date, progress is limited, since detailed mechanistic studies of the underlying PCET reactions at metal oxide surfaces are challenging. This is due to the often inhomogeneous structure of these materials, the lack of understanding of the true active PCET site(s), as well as the lack of suitable analytical techniques to study these complex materials.^5,13–18^ One promising alternative is the use of molecular metal oxides, so-called polyoxometalates (POMs) as model systems with well-defined structures that enable the study of PCET at metal–oxide surfaces. POMs are ideal models for bulk metal oxides, as their structure and reactivity are comparable to their heterogeneous analogues.^17–20^ Particularly the photoactivity of titanium-containing oxo clusters has received significant interest due to their unique structure–reactivity relationships.^21–23^

Traditionally, POMs have been widely studied as molecular reaction sites for selective thermochemical and photochemical C–H activations. Their chemical and electronic structure is comparable to many solid-state metal oxide surfaces, enabling realistic mechanistic studies in homogeneous solution.^17,24,25^ These promising features have been employed previously for (photo-) oxidative C–H activation of organic compounds, including alkanes, alkenes, alcohols, amines and glycols.^26–28^ However, seminal studies showed that the excited-state lifetimes of POMs are very short (*e.g.*, 30 ps for the prototype decatungstate, [W_10_O_32_]^4−^)^29^ so that classical diffusion-controlled reactions are challenging, as they typically require lifetimes in the nanosecond range.^30^ Pre-association between the substrate and the POM, *e.g. via* hydrogen bonding, has been put forward to overcome the diffusion limitation and enable C–H activation or hydrogen atom transfer reactivity.^26,31,32^ However, this concept only works under strictly water-free conditions, as water forms a stable, hydrogen-bonded hydration shell around the POM clusters. In this case, photoexcitation of the POM results in hydrogen-atom abstraction from water and formation of highly reactive hydroxyl radicals, leading to unselective radical oxidation chemistry.^33^

Recently, Ryu and co-workers demonstrated that polar intermolecular interactions and sterics can be combined to achieve site-selective C–H photoactivation using the decatungstate anion.^34^ Subsequent photophysical studies revealed that the reactive site is indeed a highly reactive (*E*_single-electron transfer_ = 2.44 V *vs.* SCE), short-lived radical species centered on a terminal oxygen atom, capable of initiating hydrogen atom transfer (a specific case of PCET)^35^ for a range of C–H bonds.^36^ This concept was exploited by MacMillan and co-workers who coupled photochemical decatungstate-based C–H activation with copper-catalyzed C–C bond formation to selectively introduce CF_3_ groups for the late-stage functionalization of a variety of organic substrates.^37^

While these studies show that tremendous progress has been made in the scope and application of POM-based (photo-) chemical C–H activation, to-date the underlying PCET steps which govern (photo-) reactivity and selectivity are largely unexplored. In the field of polyoxovanadates, Matson and co-workers have reported seminal studies which demonstrated a mechanistic shift from concerted PCET in homometallic polyoxovanadate-alkoxides to a stepwise pathway proceeding *via* electron transfer followed by a rate-limiting proton transfer (ET-PT) in titanium-doped polyoxovanadate–alkoxides.^18,20^ Very recently, Matson and colleagues expanded their studies to polyoxomolybdates^38^ and polyoxotungstates^39,40^ and demonstrated that PCET is a ubiquitous mechanism in POM redox-reactivity and catalysis. In related work, Sartorel and co-workers demonstrated that light-driven water-oxidation on a ruthenium-functionalized polyoxotungstate proceeds by sequential PCET steps and emphasized that the reaction environment (*e.g.*, the type and concentration of pH-buffer) is intricately linked to the PCET dynamics.^41^ Also, Mizuno and co-workers demonstrated that alcohol pre-coordination to a structural vacancy in a lacunary POM polyoxotungstate results in generation of new electronic absorptions, enabling light-driven multi-electron transfer chemistry.^42^

Following these inspirational studies, we became interested in studying light-induced PCET processes in metal-functionalized polyoxotungstates.^33^ As a model system, we identified the mono-titanium functionalized Keggin anion [(TiOH)PW_11_O_39_]^4−^ (hereafter: {TiOH})^43^ to explore and rationalize the reactivity of photoactive, reducible d^0^ metal oxides such as TiO_2_ or WO_3_. As a model reaction, we focused on the selective benzyl alcohol (BnOH)-to-benzaldehyde oxidation, which is challenging as formation of benzoic acid or higher oxidation products is difficult to prevent under (photo-) oxidative conditions. This is due to the low binding affinity and low product selectivity when using classical photocatalysts such as TiO_2_, which consequently often leads to undesired side reactions.^44–47^

Here, we report that {TiOH} can overcome these challenges by selective pre-coordination of BnOH to the Ti(iv) center, resulting in a new species with unique photophysical properties. Irradiation of this species in the visible region initiates selective benzyl alcohol-to-benzaldehyde oxidation. Comprehensive theoretical, photophysical and spectroscopic studies explore the role of light-induced PCET for selective C–H activation and alcohol oxidation at polyoxometalate photocatalysts, thus opening new avenues to selective molecular photooxidation catalysts.

## Results and discussion

### Synthesis and initial characterization

**Fig. 1:**
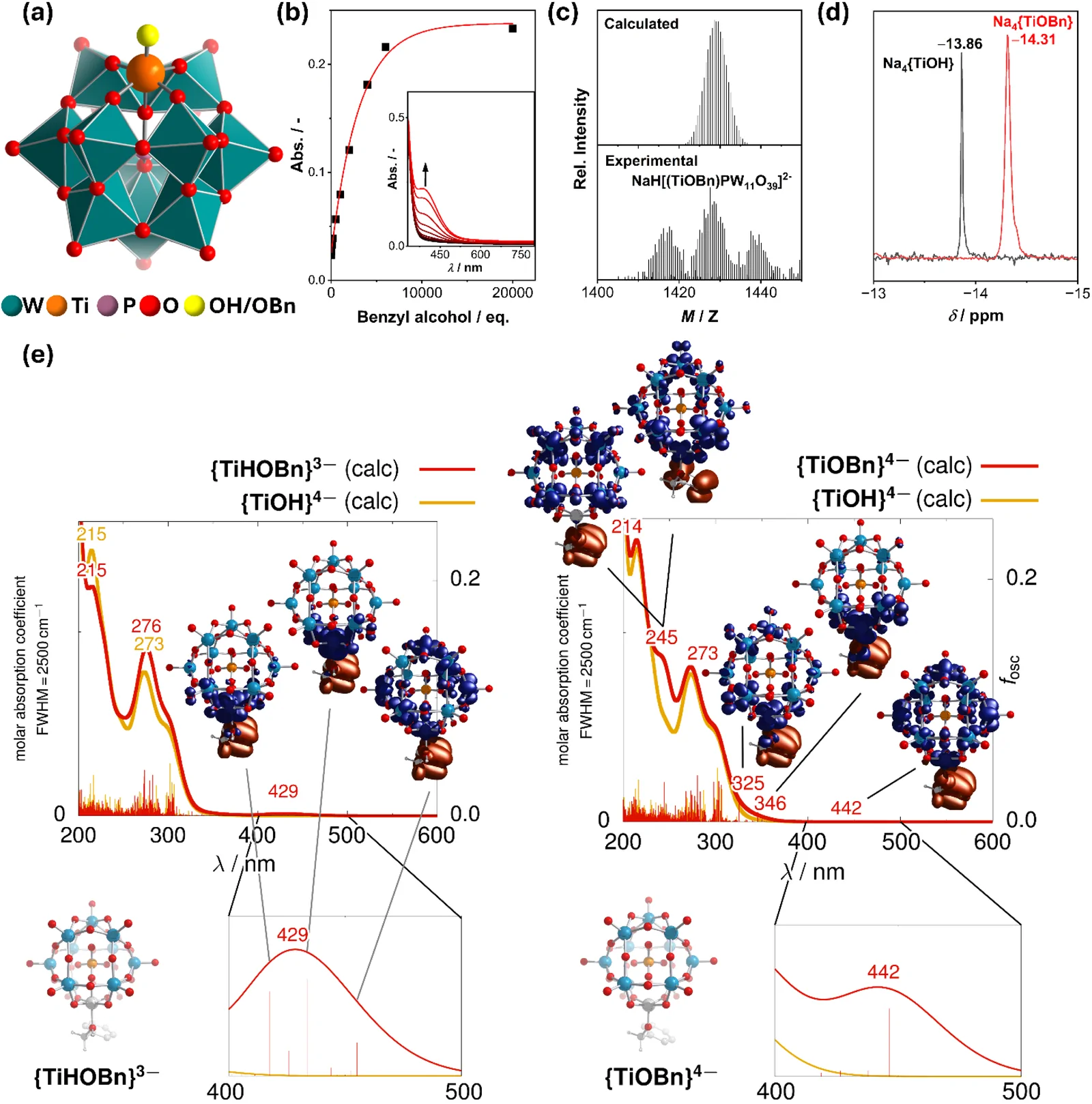
(a) Illustration of the [(TiOH)PW_11_O_39_]^4−^ cluster. (b) UV-vis spectroscopic monitoring of the characteristic increase in absorption at 425 nm, indicating the binding of benzyl alcohol to {TiOH} and formation of {TiOBn}, the red line is a guide for the eye. Inset: UV-vis absorption spectra of aqueous {TiOH} solutions upon addition of increasing equivalents of benzyl alcohol. (c) Calculated and observed isotopic patterns from high resolution negative-ion mode ESI mass spectrum of Na_4_{TiOBn}, solvent: water. (d) ^31^P-NMR spectra of Na_4_{TiOH} (black) and Na_4_{TiOBn} (red), solvent: water. (e) UV-vis spectrum for {TiHOBn} (left) and {TiOBn} (right), in red color, computed at the *r*^2^SCAN-3c level; the yellow trace shows the UV-vis spectrum of {TiOH} for comparison. Difference density plots are shown for selected transitions characteristic to {TiHOBn} and {TiOBn}; plot isovalue at 0.001 *a*_0_^−3^ with transitions from red to blue color. The low-energy LMCT bands at 429 nm and 442 nm are shown as insets.

The mono-titanium substituted Keggin anion Na_4_[(TiOH)PW_11_O_39_] (=Na_4_{TiOH}, Fig. 1a) was synthesized according to a modified literature procedure,^43^ and fully characterized (see SI, Section 2 for synthetic and analytical details). To explore the principal interactions between {TiOH} and BnOH, we followed the reaction of the cluster with increasing amounts of benzyl alcohol in aqueous citrate buffer (0.1 M, pH 5.75). Note that a buffer solution was used to prevent any undesired Brønsted acid-base reactions at the cluster or benzyl alcohol. Also note that for solubility reasons, the benzyl alcohol was introduced as a toluene solution, resulting in a biphasic water/toluene system where benzyl alcohol diffuses from the toluene to the aqueous phase (for details see SI, Section 2.3). The reaction between benzyl alcohol and {TiOH} was visually observed by a color change from colorless to bright yellow, and UV-vis-NIR spectroscopy showed the formation of an intense absorption band with an absorption maximum at *λ*_max_ = 425 nm (Fig. 1b). The observed spectral features suggest the coordination of benzyl alcohol to the Ti-binding site of {TiOH}. Notably, this reaction occurs in the aqueous phase, which is highly unusual for POMs, where binding of aquo or hydroxo ligands is typically favoured over binding of alcohols.^48^ In fact, to the best of our knowledge, this is the first example of an alcohol binding to a POM under aqueous conditions.

To further explore the experimental UV-vis spectroscopic features, we used time-dependent density functional theory (TD-DFT) to calculate the electronic transitions for [(TiHOBn)PW_11_O_39_]^3−^ (={TiHOBn}) and [(TiOBn)PW_11_O_39_]^4−^ (={TiOBn}), and compared them with the calculated UV-vis spectra of {TiOH}, see Fig. 1e. Both {TiOBn} and {TiHOBn} feature characteristic ligand-to-metal charge-transfer (LMCT) transitions in the region between 400 and 450 nm, assigned to benzyl alcohol-to-POM-metal center transitions. This is in line with the observed increase in visible light absorption, and the observed color change from colorless to yellow. Additional LMCT absorptions in the UV range around 245 nm were identified for the deprotonated, alkoxide-containing species {TiOBn}. Note that excellent agreement between computed and experimental spectral features was also observed for the reference compounds [PW_12_O_40_]^3−^ and the titanium-functionalized species {TiOH} (SI, Fig. S16).

To verify the coordination of the benzyl alcohol to the cluster, we performed a series of mechanistic and analytical experiments: ^31^P-NMR spectra of the reaction solution were recorded before and after the addition of benzyl alcohol (Fig. 1d). Na_4_{TiOH} shows a single signal at −13.9 ppm, due to the central phosphate template. Upon addition of 8000 equivalents of benzyl alcohol, this signal shifts to −14.3 ppm, indicating the formation of a single new phosphorus-containing cluster species. Also, we used high-resolution electrospray ionization mass spectrometry (HR-ESI MS) to study BnOH binding to the POM. Analysis of an aqueous solution containing Na_4_{TiOH} (1 mM) and benzyl alcohol (8000 equivalents, 8 M) shows the presence of species which fit the isotopic envelope for NaH[(TiOBn)PW_11_O_39_]^2−^ (observed: *m*/*z* 1429.125, calcd: *m*/*z* 1429.111, Fig. 1c). This provides further evidence for the formation of a benzyl alcohol-bound Ti-Keggin species. Alcohol coordination is most likely to occur at the Lewis-acidic Ti(iv) site which features a labile hydroxide ligand, while the W(vi) centers contain oxo ligands which are not expected to undergo ligand exchange under the given reaction conditions.^49^ In addition, the presence of excess of BnOH is a likely factor which drives the alcohol coordination to the POM in a dynamic equilibrium.

Next, we set out to determine whether the benzyl alcohol is bound as a protonated alcohol, or a deprotonated alkoxide species. To this end, we performed pH measurements of a 1 mM Na_4_{TiOH} solution in water (initial pH = 5.2). Upon addition of 8000 equivalents of benzyl alcohol, we observe a change in the pH value to 3.6. For the reference experiment where the identical amount of BnOH was added to pure water, we also observed a drop in pH value (to pH = 4.7). This indicates that binding of the benzyl alcohol occurs *via* a deprotonation route, where a proton is transferred to the Ti–OH hydroxide ligand, which is eventually released as water. The alternative reaction mechanism where the Ti–OH ligand is simply exchanged with a (protonated) BnOH ligand would result in release of OH^−^ into the solution, resulting in a theoretical final pH value of *ca.* 11, which is not observed experimentally. This interpretation is further supported by DFT calculations which show that coordination of benzyl alcohol *via* deprotonation and release of water is exergonic (Δ_r_*G* = −3.1 kcal mol^−1^) and energetically favoured over hydroxide-for-benzyl alcohol ligand exchange (Δ_r_*G* = 56.2 kcal mol^−1^), see SI for details. This is in line with coordination chemistry considerations which indicate that the alkoxide is a much stronger ligand compared with the protonated alcohol.^50^ In sum, our experimental and theoretical data suggest the formation of the species [(TiOBn)PW_11_O_39_]^4−^, hereafter: {TiOBn}. Note that despite significant efforts, crystallization of this compound was not possible, so this study reports no single-crystal X-ray diffraction data.

To isolate {TiOBn} from solution, a cation metathesis was performed by reacting aqueous solutions of Na_4_{TiOBn} with tetra-*n*-butylammonium bromide (*n*Bu_4_NBr, see SI for experimental details). This resulted in precipitation of a yellow solid, which was characterized *via* ATR-IR (SI, Fig. S2), cyclic voltammetry (SI, Fig. S13) and ^1^H-NMR spectroscopy (SI, Fig. S4). Based on this analysis, we identified the compound as (*n*Bu_4_N)_4_[(TiOBn)PW_11_O_39_] (hereafter: (*n*Bu_4_N)_4_{TiOBn}). Specifically, ^1^H DOSY NMR spectroscopy of (*n*Bu_4_N)_4_{TiOBn} in MeCN demonstrated the persistent binding of OBn^−^ to the POM in organic solution: while BnOH shows a diffusion coefficient of 3.94 × 10^−5^ cm^2^ s^−1^, (*n*Bu_4_N)_4_{TiOBn} gave a lower diffusion coefficient of 1.30 × 10^−5^ cm^2^ s^−1^. This shows that the OBn^−^ species diffuses as a larger compound, and the diffusion coefficient magnitude is in line with previous reports on POM diffusion coefficients (SI, Fig. S8).^51^ We performed a similar metathesis route to also access the organo-soluble reference compound (*n*Bu_4_N)_4_{TiOH} (=(*n*Bu_4_N)_4_[(TiOH)PW_11_O_39_]) which was obtained by reaction of aqueous solutions of {TiOH} with excess *n*Bu_4_NBr, see SI, Section 2 and 3 for synthetic and analytical details.

Next, we performed preliminary analyses on how the oxidation of BnOH is changed by coordinative pre-association to {TiOH}. To this end, we performed cyclic voltammetry studies in water-free acetonitrile. First, we studied the pure BnOH reference (in the absence of the POM), which shows oxidation waves at 2.0 V *vs.* Fc^+^/Fc and 2.4 V *vs.* Fc^+^/Fc. In contrast, for a mixture of BnOH and (*n*Bu_4_N)_4_{TiOH}, we observe two irreversible oxidation waves (assigned to BnOH oxidation) at 0.45 V and 0.75 V *vs.* Fc^+^/Fc, *i.e.*, at significantly lower potentials than the POM-free reference experiment (SI, Fig. S13). This indicates that pre-coordination of the BnOH to the POM can facilitate alcohol oxidation and forms the basis for the subsequent photochemical studies.

### Benzyl alcohol oxidation studies

**Fig. 2:**
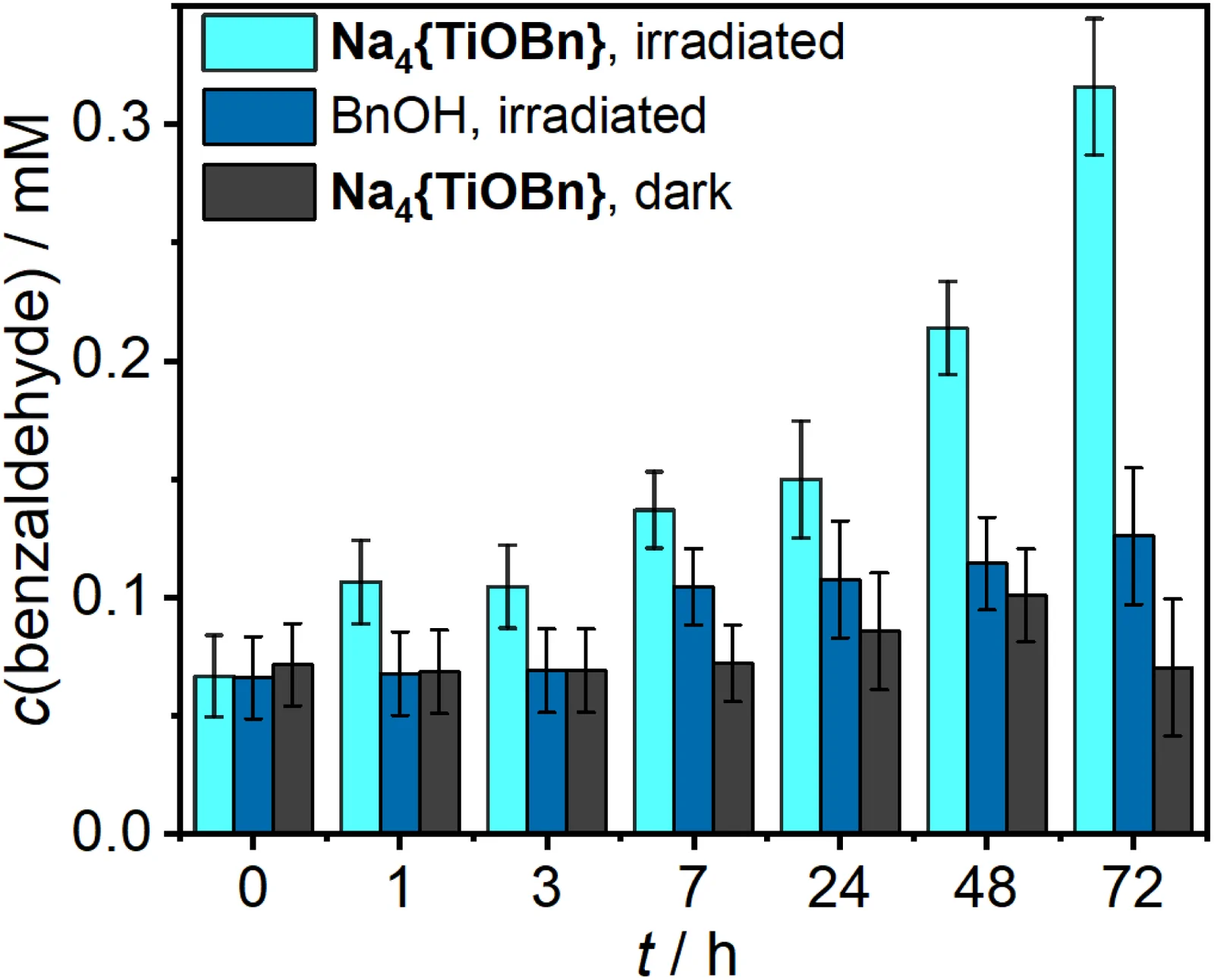
{TiOBn}-induced visible light photooxidation of BnOH to benzaldehyde, showing the benzaldehyde concentration increase over time. Shown are data for reaction containing {TiOBn} under irradiation (light blue), the POM-free reference reaction under irradiation (blue) and the {TiOBn}-containing reference reaction without irradiation (dark grey). Conditions: solvent biphasic reaction (aqueous citrate buffer (0.1 M, pH = 5.75)/toluene, for details see SI), [{TiOH}] = 25 mM, [BnOH] = 1 M; LED irradiation wavelength: 470 nm.

Based on the data above, we hypothesized that {TiOBn} could be capable of visible light-induced benzyl alcohol oxidation in water by photoexcitation of the characteristic vis-LMCT bands (Fig. 1e). To this end, the VIS-photoactive compound {TiOBn} was generated *in situ* by coordination of BnOH to Na_4_{TiOH}. Specifically, a solution of Na_4_{TiOH} (25 mM) in aqueous citrate buffer (0.1 M, pH 5.75, 1 mL) and a toluene solution of benzyl alcohol (1 M, 5 mL) were combined, resulting in a biphasic system which provides a reservoir phase for both the reagent (BnOH) and the product (benzaldehyde). The biphasic system was irradiated with a 470 nm LED at ambient conditions over a period of 72 hours. The concentration change of benzaldehyde in the organic phase was quantified by high-pressure liquid chromatography (HPLC). Each measurement was carried out in triplicate. Note that reference experiments showed that when the reaction was not irradiated, or when no POM was present in solution, only small amounts of benzaldehyde were detected, thereby demonstrating that the observed reactivity is both POM-dependent and light-dependent, and not simple autoxidation of benzyl alcohol (see Fig. 2).^52^ In contrast, for the Na_4_{TiOBn}-containing reaction, we observe continuous increase in benzaldehyde formation over the course of 72 h irradiation (Fig. 2).

Post-reaction studies by UV-vis absorption spectroscopy (after 72 h of irradiation) indicate that the benzyl alcohol-to-metal LMCT bands have disappeared, and instead show the emergence of a characteristic POM-based intervalence charge-transfer (IVCT) band between 600 nm to 850 nm, assigned to the formation of delocalized W(v) centers expected for a reduced Na_4_{TiOH} species (see SI, Fig. S10).^49^ This is in line with the expected POM reduction during benzyl alcohol oxidation. ^13^C-NMR spectroscopy (SI, Fig. S12) shows no observable amount of benzoic acid in the aqueous solution or the toluene phase, which is in line with formation of benzaldehyde as the sole product.

### UV-vis transient absorption spectroscopy

To further identify the nature of the reactive species, we performed UV-vis pump-probe transient absorption (TA) spectroscopy for acetonitrile solutions of (*n*Bu_4_N)_4_{TiOH} and (*n*Bu_4_N)_4_{TiOBn}. Note that (*n*Bu_4_N)_4_{TiOBn} was prepared *in situ* by addition of 8000 equivalents of benzyl alcohol to a 2.5 mM solution of (*n*Bu_4_N)_4_{TiOH} in acetonitrile. (*n*Bu_4_N)_4_{TiOH} was irradiated with pump pulses at 267 nm to excite the oxygen-to-metal charge transfer band, while (*n*Bu_4_N)_4_{TiOBn} was irradiated with pump pulses at 400 nm to excite into the new BnO-to-POM charge transfer band (Fig. 1b). The time resolution of these experiments was about 150 fs.

**Fig. 3:**
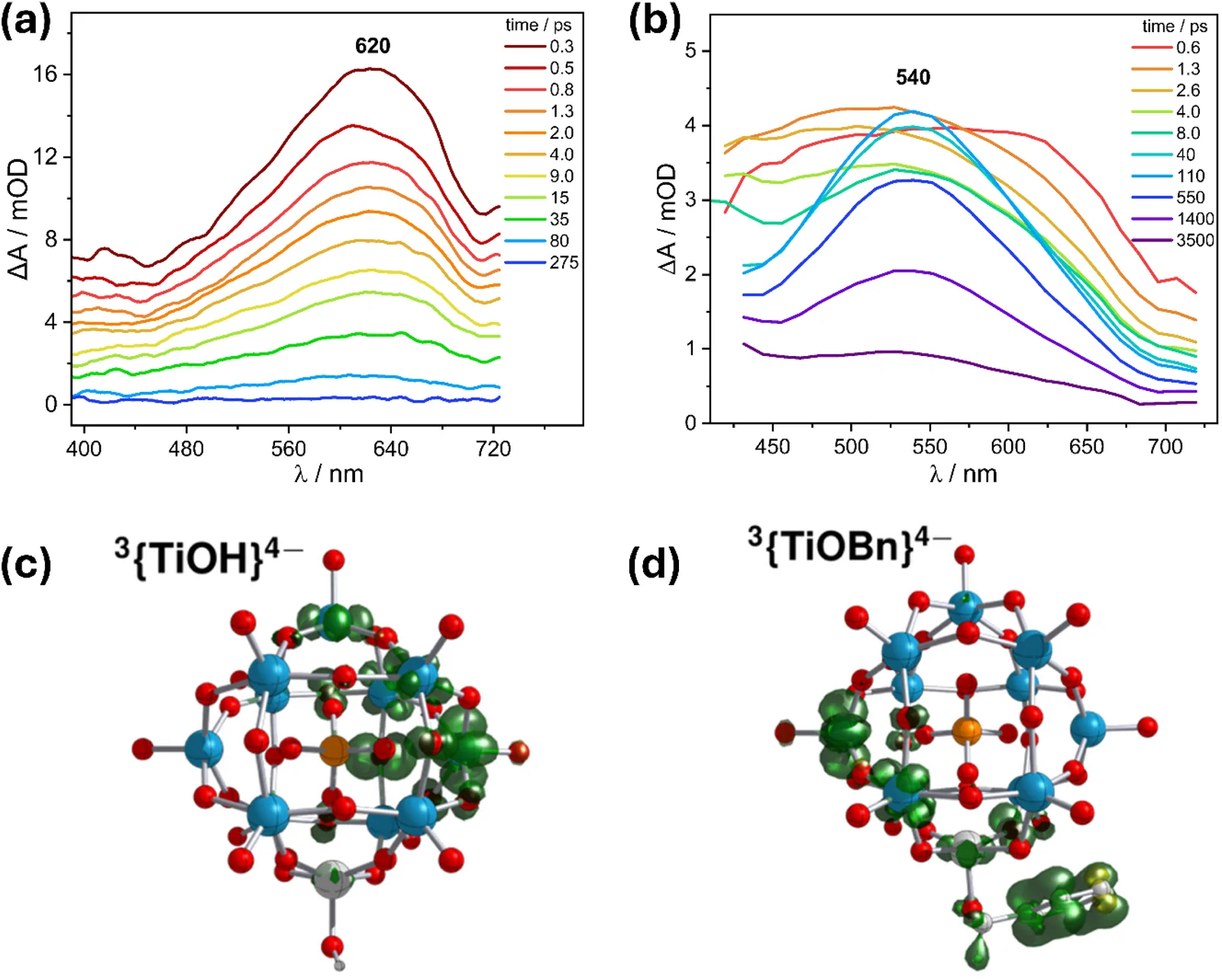
Transient absorption difference spectra of (a) (*n*Bu_4_N)_4_{TiOH} (excitation wavelength 267 nm) and (b) (*n*Bu_4_N)_4_{TiOBn} (excitation wavelength 400 nm) at pump-probe delay times as indicated; solvent MeCN, *T* = 295 K. Time traces for selected probe wavelengths are presented in the SI, Fig. S18 and S19. (c and d) Spin densities (isovalue at 0.005 *a*_0_^−3^) for triplet states of {TiOH}^4−^ (c) and {TiOBn}^4−^ (d) at relaxed molecular geometries; adiabatic singlet-triplet excitation energies, Δ*E*(S/T) = 63 kcal mol^−1^ (for {TiOH}^4−^) and 57 kcal mol^−1^ (for {TiOBn}^4−^); structural relaxation of the triplet state from the related singlet geometry corresponds to 6 kcal mol^−1^ (for {TiOH}^4−^) and 10 kcal mol^−1^ (for {TiOBn}^4−^).

When (*n*Bu_4_N)_4_{TiOH} is excited at 267 nm, a broad TA band with a maximum at 620 nm is instantly produced (Fig. 3a). It closely resembles the spectrum of a singly reduced tungstate cluster, which exhibits a broad IVCT transition centred at approximately 600 nm.^53^ The transition is caused by the presence of reduced tungsten centres, enabling the transfer of an electron between W(v) and W(vi). The 267 nm photo-induced appearance of this IVCT band is consistent with literature, where the primary excitation in the polyoxometalate is described by an oxygen-to-metal charge-transfer producing formally a W(v) metal centre and an oxygen centred radical.

In line with previous studies, the POM charge-separated state is short-lived and undergoes fast recombination within 100 ps.^25,26^ Interestingly, a large range of timescales is involved in this process, as the decay of the IVCT band in Fig. 3a can be described by three time constants: *τ*_1_ = 0.24 ± 0.05 ps, *τ*_2_ = 2.0 ± 0.2 ps, and *τ*_3_ = 44 ± 5 ps, with relative amplitudes of approximately 30%, 30%, and 40%, respectively (SI, Fig. S18). This can be explained assuming that after excitonic excitation into the singlet manifold, rapid intersystem crossing and valence trapping occur, thereby producing W(v) sites at various distances *r* to the oxygen acceptor. Such a scenario is consistent with the calculated electronic structure of the T_1_ state showing incomplete delocalisation of the spin density over the cluster (Fig. 3c). Thus, if the thermal electron hopping rate from W(v) to W(vi) is slower than the recombination rate, this would result in a distribution of lifetimes, given that the electron transfer rate is proportional to exp(−*β r*),^54^ where *β* is a characteristic inverse length determined by the overlap of the wavefunctions of the donor and acceptor moieties. For non-conjugated molecular systems *β* values are typically ≈1 Å^−1^.^55^ With electron–hole distances of 2–7 Å estimated from the calculated {TiOH} structure, the resulting electron transfer rate constants vary by a factor of 150, which is in excellent agreement with our observed lifetime differences. Complementary measurements on the reference cluster (*n*Bu_4_N)_3_[PW_12_O_40_] {PW_12_} (SI, Fig. S20) show a similar UV-vis absorption band shape as well as a distribution of lifetimes comparable to those of {TiOH} (SI, Fig. S21), thereby providing further support for the photophysical behaviour proposed above.

The excited state dynamics of {TiOBn} are distinctly different compared to {TiOH} (Fig. 3b). Following excitation at 400 nm, a broad excited state absorption band covering the spectral range from 400 nm to 700 nm is instantly formed. It decays with a time constant of *τ*_1_ = 2.5 ± 0.5 ps before, with some delay, a sharper absorption feature with a maximum at *λ*_max_ = 540 nm emerges (time traces with multi-exponential fits are presented in the SI (Fig. S18)). The sharpening of the 540 nm band is associated with a time constant of *τ*_2_ = 18 ± 4 ps. The 540 nm band is rather long-lived and decays with a single time constant of *τ*_3_ = 2.0 ± 0.2 ns.

According to our DFT calculations, the optically allowed transition at 425 nm corresponds to an LMCT from the benzyl alcohol to the POM yielding a charge-separated excited state with an alkoxide radical and a one-electron reduced cluster with the electron delocalized throughout the POM. The calculated electronic structure of the T_1_ state suggests that after rapid intersystem crossing, the electron is localized on individual WO_6_ units (Fig. 3d). The resulting mixed-valence configuration results in a W(v)–W(vi) IVCT band consistent with the broad TA spectra observed instantly after cluster excitation. The following decay of the IVCT band and formation of the 540 nm species with its significantly narrower absorption band is compatible with final electron localization on the Ti center thereby generating a Ti(iii) species. This assignment is supported by studies on titanium-oxo clusters, which were photochemically reduced to contain Ti(iii) species in the presence of alcohols as sacrificial electron donors.^56,57^ In these systems, formation of Ti(iii) centers results in comparatively narrow absorption bands, closely matching the 540 nm spectral feature observed for {TiOBn}.^56,57^ Based on our analyses and the literature precedent, we tentatively assign the 540 nm band to a Ti(iii) d–d transition. The fact that this band requires almost 70 ps (associated time constant *τ*_2_ = 18 ps) to completely emerge indicates that after electron localization on the Ti center, major structural reorganization in the vicinity of the metal takes place.

Combining the observations with the known literature we assign the observed 540 nm excited state absorption feature to a formally reduced titanium(iii) cation with a semi-strictly localized electron. The presence of an oxidized benzyl alcohol radical is consistent with a transient absorption observed at around 450 nm, which is characteristic for aromatic radicals.^58^

It should be noted that the intensity of the TA signal slowly decreased with each measurement cycle which is why for the final measurement a flow cell with large reservoir volume was used. This indicates that one of the decay pathways contributing to the 2.0 ns excited state lifetime is the reaction of benzyl alcohol to benzaldehyde.

### Computational analysis of the POM-mediated benzyl alcohol oxidation

**Fig. 4:**
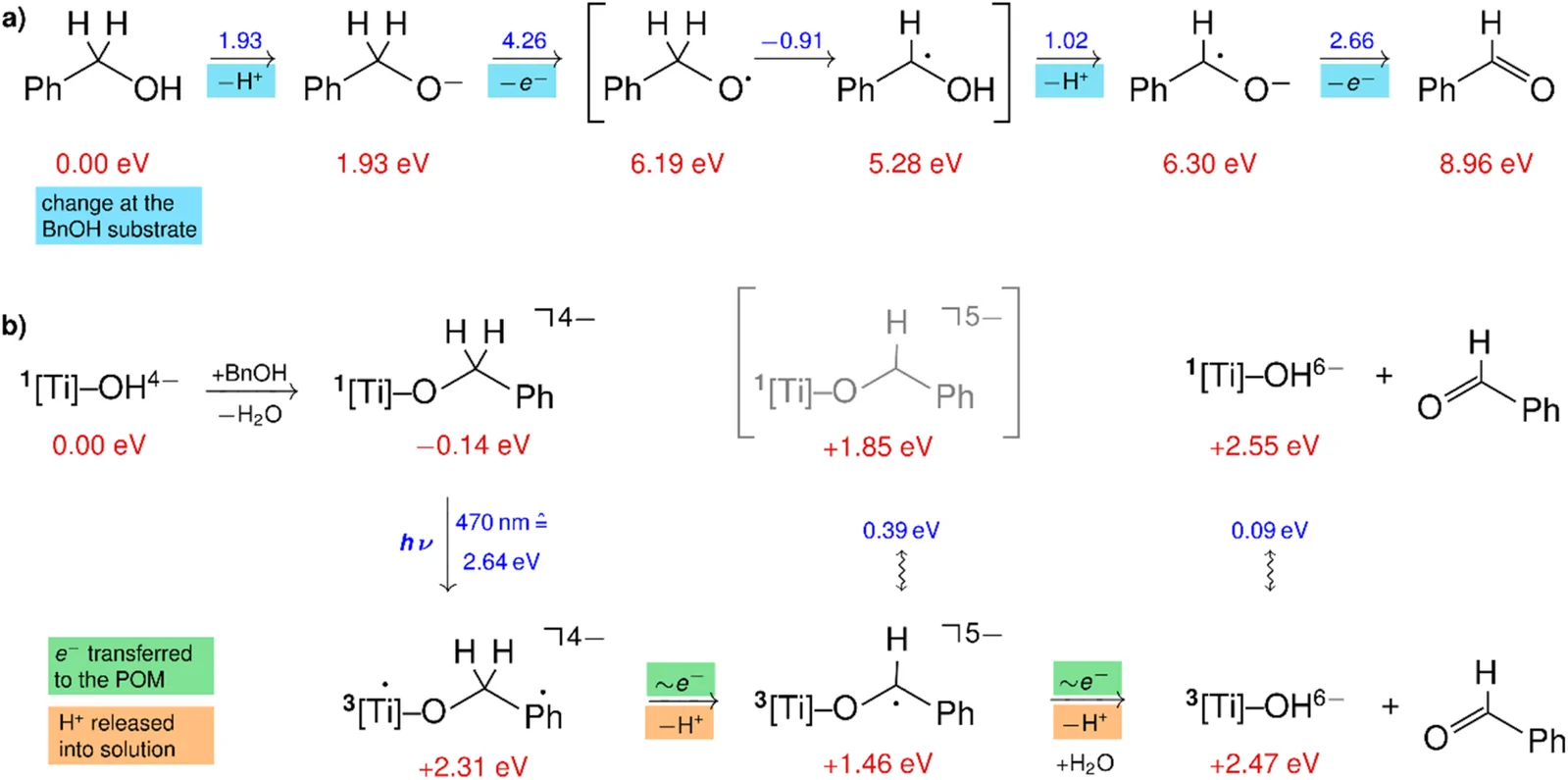
Relative Gibbs energies (a) for the oxidation of benzyl alcohol to benzaldehyde, and (b) for the POM-assisted redox reaction starting from {TiOH}^4−^ and benzyl alcohol. Energies are given in eV, individual steps in blue color, relative energies for intermediates in red color. The Gibbs energy value of −11.55 eV for solvation of the proton in water was taken from the estimate of Rossini and Knapp,^59^ and is included in the corresponding deprotonation steps.

Next, we used DFT computations to study the 2-proton/2-electron oxidation of benzyl alcohol to benzaldehyde. In the gas phase, the computed hypothetical oxidation of BnOH to benzaldehyde involves several charge separation events and requires ∼9 eV (Fig. 4a). Turning to the benzyl alcohol oxidation with a POM mediated reaction (Fig. 4b), the proposed mechanism involves three distinct steps: first, the hydroxyl ligand of {TiOH}^4−^ is replaced by BnOH accompanied by release of water, forming {TiOBn}^4−^. Next, photoexcitation of {TiOBn}^4−^ results in generation of a triplet state which can undergo electron transfer (from BnO to the POM) and deprotonation to form a charge-separated species. This species then undergoes a second electron transfer from the BnO radical to the POM, resulting in formation of the benzaldehyde product. The terminal Ti–OH group is then reformed by release of the benzaldehyde, coordination of water and release of a proton. In sum, the process therefore is a 2-proton/2-electron oxidation of benzyl alcohol to benzaldehyde (Fig. 4b). Our computations show that this POM-mediated mechanism reduces the energetic demands by ∼6.5 eV. Also, note that the triplet manifold competes with an energetically slightly less favoured singlet route (Fig. 4b). The spin densities of the triplet intermediates imply formation of W(v) centres along with electron holes on the BnO ligand.

### Nonaqueous Pourbaix analysis of the POM PCET capabilities

**Fig. 5:**
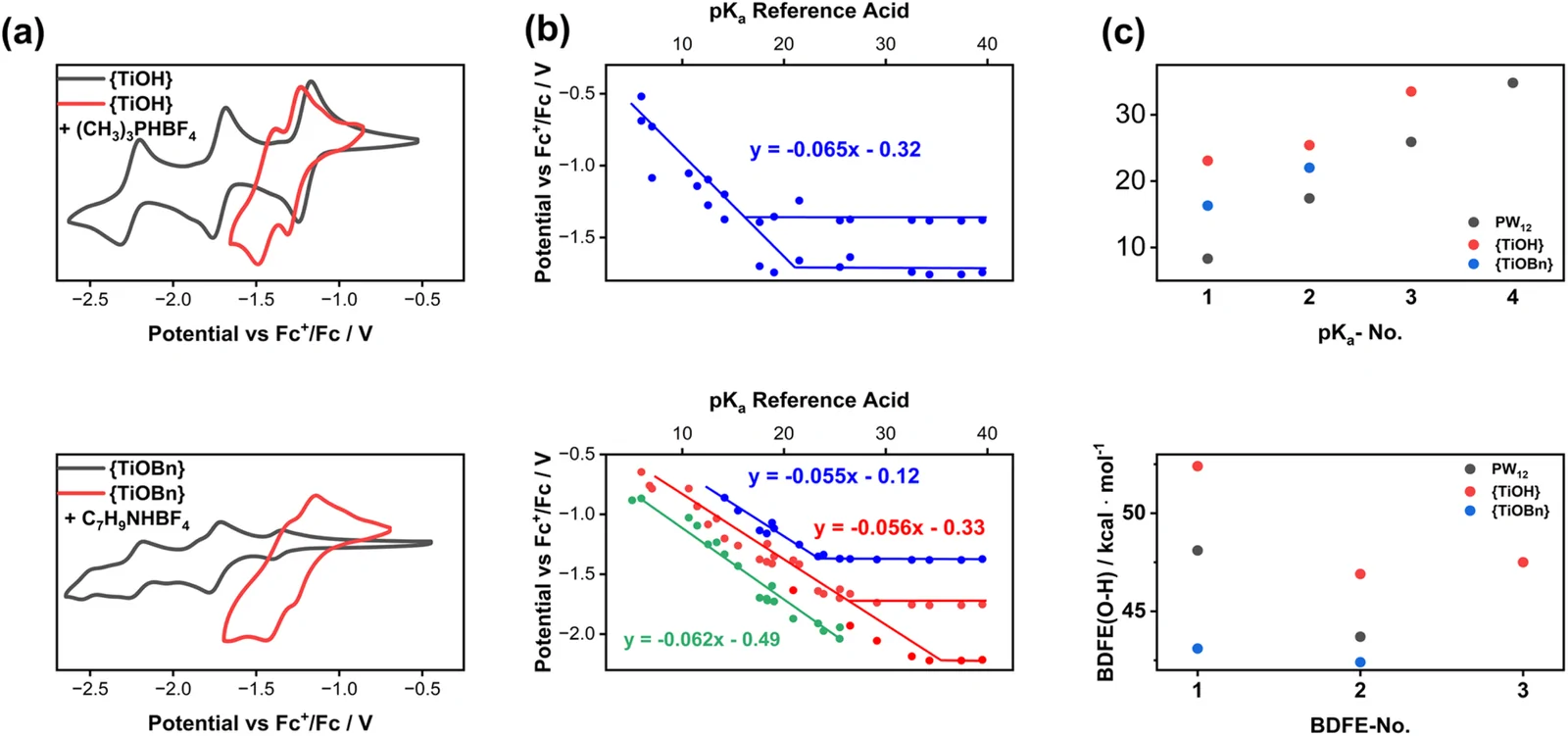
(a) Top: cyclic voltammogram of 1 mM (*n*Bu_4_N)_4_{TiOH} in acetonitrile (p*K*_a_(MeCN) = 39.5) (black) and in presence of 4 mM trimethylphosphonium tetrafluoroborate ((CH_3_)_3_PHBF_4_)(p*K*_a,MeCN_ ((CH_3_)_3_PHBF_4_) = 15.5) (red) in acetonitrile (containing 0.1 M (*n*Bu_4_N)PF_6_, scan rate: 100 mV s^−1^, internal reference: Fc^+^/Fc). Bottom: cyclic voltammogram of 1 mM (*n*Bu_4_N)_4_{TiOBn} in acetonitrile (p*K*_a_(MeCN) = 39.5) (black) and in presence of 4 mM 2,6-lutidinium tetrafluoroborate (C_7_H_9_NHBF_4_)(p*K*_a,MeCN_ (C_7_H_9_NHBF_4_) = 14.2) (red) in acetonitrile (containing 0.1 M (*n*Bu_4_N)PF_6_, scan rate: 100 mV s^−1^, internal reference: Fc^+^/Fc) (b) Top: potential-p*K*_a_ diagram of (*n*Bu_4_N)_4_{TiOH}. Bottom: potential-p*K*_a_ diagram of (*n*Bu_4_N)_4_{TiOBn}. (c) Top: comparison of p*K*_a_ values calculated for PW_12_ (black), (*n*Bu_4_N)_4_{TiOH} (red) and (*n*Bu_4_N)_4_{TiOBn} (blue). Bottom: calculated BDFE(O–H) for PW_12_ (black), (*n*Bu_4_N)_4_{TiOH} (red) and (*n*Bu_4_N)_4_{TiOBn} (blue).

The (*n*Bu_4_N)_4_{TiOH}-driven photooxidation of benzyl alcohol to benzaldehyde is a net transfer of two protons and two electrons. To explore the role of the POM in proton and electron storage^49,60^ we studied the reduction and protonation of {TiOH} and {TiOBn} by electrochemistry and nonaqueous Pourbaix analysis in acetonitrile. This approach is inspired by recent work by Matson and coworkers who used a similar experimental approach to study reduction/protonation of the Keggin anion {PW_12_},^17^ other tungstate POMs,^40^ doped polyoxotungstates,^39,61–63^ polyoxovanadates^16,20^ and -molybdates.^38^ These studies showed that in acetonitrile, {PW_12_} shows four reversible 1e^−^ redox events at −0.70 V, −1.2 V, −1.9 V and −2.5 V *vs.* Fc^+^/Fc (Fig. 5). For (*n*Bu_4_N)_4_{TiOH}, we observe three reversible 1e^−^ redox events at −1.3 V, −1.8 V and −2.2 V *vs.* Fc^+^/Fc (Fig. 5), while (*n*Bu_4_N)_4_{TiOBn} shows three reversible redox processes at −1.4 V, −1.8 V and −2.2 V *vs.* Fc^+^/Fc. Based on redox-chemical considerations and comparison with the literature, we hypothesized that the observed redox-transitions are due to W(vi/v) redox couples.^17^ Also, we used DFT to calculate the first and second reduction of {TiOBn} and found satisfactory matches (Δ*E* < 0.25 V, Table 1) with the experimental data, thereby providing further evidence that the redox transitions are based on W(VI/V) redox couples (for details, see SI). Based on this data, we note that {TiOH} and {TiOBn} require more negative redox potentials for the first reduction compared with {PW_12_}, which is expected based on the increased anionic charge.

**Table 1:** Experimental and computed redox potentials in V relative to Fc+/Fc for {TiOH}*^n^*^−^ and {TiOBn}*^n^*^−^, *n* = 4–7. DFT computations performed at the *r*^2^SCAN-3c level

Species	Redox potential (exp.) [redox potential (calcd.)]/*V vs.* Fc^+^/Fc	Species	Redox potential (exp.) [redox potential (calcd.)]/*V vs.* Fc^+^/Fc
{TiOH}^4−/5−^	−1.3 [−1.1]	{TiOBn}^4−/5−^	−1.4 [−1.1]
{TiOH}^5−/6−^	−1.8 [−1.7]	{TiOBn}^5−/6−^	−1.8 [−1.7]
{TiOH}^6−/7−^	−2.2 [−2.0]	{TiOBn}^6−/7−^	−2.2 [−2.0]

Next, we analyzed the PCET thermodynamics of the POMs by non-aqueous Pourbaix analysis in MeCN. This allows us to determine p*K*_a_ values as well as bond dissociation free energies (BDFE(O–H)) for (*n*Bu_4_N)_4_{TiOH} and (*n*Bu_4_N)_4_{TiOBn}, and thereby explore the POM behaviour upon reduction and protonation.^64^ First, we showed that all slopes of the p*K*_a_-dependent regions in the Pourbaix diagram of (*n*Bu_4_N)_4_{TiOH} are close to the theoretical value of −59 mV per p*K*_a_ unit expected for 1e^−^/1H^+^ PCET steps (Fig. 5b; see SI for details). The second and third redox processes converge into a 2e^−^/2H^+^ process (see SI, eqn (2)), while the fourth redox process – observed for acids with p*K*_a_ < 25 – exhibits a slope of −62 mV per p*K*_a_ unit (Fig. 5b). In sum, these findings demonstrate that (*n*Bu_4_N)_4_{TiOH} can undergo four 1e^−^/1H^+^ PCET processes in the electrochemically accessible solvent window and in the p*K*_a_ range studied. In contrast, for (*n*Bu_4_N)_4_{TiOBn} we only observe one 1e^−^/1H^+^ PCET which converges into a 2e^−^/2H^+^ process with an average slope of −65 mV p*K*_a_^−1^-unit (Fig. 5b, and SI). The third redox process at −2.22 V *vs.* Fc^+^/Fc disappears upon addition of H^+^-donors, indicating that the BnO ligand restricts the cluster's redox activity (See Fig. 5b).

The Pourbaix analysis also provided the p*K*_a_ values for each redox state of the POMs studied, as shown in Fig. 5c (for details, see SI). Based on this analysis, (*n*Bu_4_N)_4_{TiOBn} shows a higher acidity compared with (*n*Bu_4_N)_4_{TiOH}. This might be due to partial protonation of the cluster shell during the binding of BnOH. Similar behaviour has been reported for {PW_12_} in presence of certain acids, *e.g.* 2,6-lutidinium tetrafluoroborate.^17^ Finally, we used the Bordwell equation to calculate the O–H BDFE for the reduced (*n*Bu_4_N)_4_{TiOH} and (*n*Bu_4_N)_4_{TiOBn} (Fig. 5c) using the experimentally obtained redox potentials and p*K*_a_ values from the Pourbaix analysis:^65,66^BDFE (*X* − *H*) = 1.37 p*K*_a_ (*XH*) + 23.06 *E*° + *C*_g,sol_where *C*_g,sol_ is a solvent dependent empirical constant, for acetonitrile: 52.6 kcal mol^−1^. Based on this analysis, for (*n*Bu_4_N)_4_{TiOH} we find BDFEs of 52.4 kcal mol^−1^, 46.9 kcal mol^−1^ and 47.5 kcal mol^−1^, while for (*n*Bu_4_N)_4_{TiOBn}, we find 43.1 kcal mol^−1^ and 42.4 kcal mol^−1^. Note that in comparison with the parent compound {PW_12_}, the BDFEs of (*n*Bu_4_N)_4_{TiOH} are up to 10 kcal mol^−1^ higher (52.3 kcal mol^−1^*vs.* 42.4 kcal mol^−1^).^17^ This transition-metal-induced strengthening of the surface O–H bond reflects studies by Matson and co-workers for heteroatom-doped Keggin-polyoxotungstates, but here it arises from a Lewis-acidic Ti(iv) center, rather than from redox-active dopants that gate electron and proton transfer in these related systems.^39,61^ Therefore the BDFEs of (*n*Bu_4_N)_4_{TiOH} indicate a higher proton affinity compared to {PW_12_} which is in agreement with the higher basicity of (*n*Bu_4_N)_4_{TiOH}. The combined analysis of BDFE values, redox potentials and p*K*_a_-values show that (*n*Bu_4_N)_4_{TiOBn} can thermodynamically host the two H^+^/e^−^-equivalents released during the oxidation of benzyl alcohol, consistent with transient proton-coupled electron storage on the POM surface in acetonitrile.

## Conclusions

In sum, this manuscript reports the unique selective photooxidation reactivity of a titanium polyoxotungstate in aqueous solvent. Experimental and theoretical analyses were combined to elucidate the pathway for the selective visible light-driven oxidation of benzyl alcohol to benzaldehyde by [(TiOH)PW_11_O_39_]^4−^. The initial key reaction step is benzyl alcohol pre-coordination to the Lewis-acidic Ti(iv) center which results in the formation of a VIS-photoactive species capable of undergoing excited-state proton-coupled electron transfer. Computational analyses reveal the energetics of this process, while ultrafast transient-absorption spectroscopic analyses reveal the processes occurring upon initial photoexcitation. In sum, both, transition metal-functionalization and benzyl alcohol coordination result in a unique alkoxide–polyoxometalate complex with unique photophysical properties and reactivity. Electrochemical Pourbaix analyses support these findings and highlight that the POM cluster can thermodynamically accommodate the redox equivalents (H^+^/e^−^) released during the photooxidation. In addition, the study outlines design principles on how these concepts can be expanded, *i.e.*, the incorporation of redox-active, Lewis-acidic metal sites featuring accessible substrate binding sites within the POM shell. In sum, this work opens new avenues for designing photoactive POMs featuring substrate binding sites for photooxidation chemistry in water as benign solvent.

## Author contributions

P. K.: conceptualization, data curation, formal analysis, investigation, methodology, validation, visualization, writing – original draft, writing – review and editing; K. S.: data curation, formal analysis, methodology, validation, visualization, writing – original draft, writing – review and editing; D. A.: data curation, formal analysis, investigation, writing – review and editing; S. B.: data curation, formal analysis, investigation, writing – review and editing; M. D.: formal analysis, validation, visualization, writing – original draft, writing – review and editing; D. S.: conceptualization, funding acquisition, methodology, resources, supervision, validation, writing – review and editing; V. K.: conceptualization, formal analysis, funding acquisition, methodology, project administration, resources, supervision, validation, writing – original draft, writing – review and editing; C. S.: conceptualization, formal analysis, funding acquisition, project administration, resources, supervision, validation, visualization, writing – original draft, writing – review and editing.

## Conflicts of interest

There are no conflicts to declare.

## Supplementary Material

SC-017-D6SC04575H-s001

## Data Availability

The data that support the findings of this study are openly available in https://zenodo.org at https://doi.org/10.5281/zenodo.20022280, reference number 20022280. Supplementary information (SI): experimental and theoretical data. See DOI: https://doi.org/10.1039/d6sc04575h.
