# Supplementary material for: Pre-association enables visible-light induced proton-coupled electron-transfer in a titanium-functionalized polyoxotungstate
Source: Chem Sci. 2026 Jul 13;17(34):16228–37. doi: 10.1039/d6sc04575h (PMC13386341; doi:10.1039/d6sc04575h)
Supplement: Supplementary file 1 [file SC-017-D6SC04575H-s001.pdf]

Supplementary Information (SI) for Chemical Science.
This journal is © The Royal Society of Chemistry 2026

Electronic Supplementary Information for

Pre-association enables visible-light induced proton-coupled electron-transfer in a titanium-functionalized polyoxotungstate

Paul Kadereit,[a] Kristin Sellmann,[a] Dayana M. G. Alban,[b] Sayan Kangsa Banik,[c] Martin Diefenbach,[b] Dirk Schwarzer,*[c] Vera Krewald,*[b] Carsten Streb*[a]

Contents

1. Instrumentation: ... 1
2. Experimental Section ... 2
   2.1 Synthesis of $Na_4[(TiOH)PW_{11}O_{38}]$ ($Na_4\{TiOH\}$) ... 2
   2.2 ESI-MS Spectrometry ... 3
   2.3 Benzyl alcohol titration ... 3
   2.5 ATR-IR spectroscopy ... 4
   2.6 Thermogravimetric analysis ... 4
   2.7 ^{1}H-NMR spectroscopy ... 5
   2.8 ESI Mass Spectrometry ... 6
   2.9 ^{1}H-DOSY spectroscopy ... 7
   2.10 UV-VIS spectroscopy after benzyl alcohol oxidation ... 8
   2.11 ^{13}C-NMR spectroscopy after benzyl alcohol oxidation ... 8
   2.12 Cyclovoltammetry of $(n\mathrm{Bu}_4\mathrm{N})_4$\{TiOBn\} ... 9
   2.13 Nonaqueous Pourbaix analysis ... 9
3. Computational Section ... 12
4. UV-Vis Transient Absorption Spectroscopy Section ... 16
5. References ... 19

1. Instrumentation:

Attenuated total Reflectance-Fourier transform Infrared Spectroscopy (ATR-FTIR) was carried out on a Bruker Alpha II FTIR spectrophotometer containing a Diamond crystal ATR unit. Signals are presented as wave numbers in cm^{-1} using the following abbreviations: vs = very strong, s = strong, m = medium, w = weak and b = broad.

Nuclear magnetic resonance spectroscopy (NMR) was recorded using a Bruker Avance Neo NMR spectrometer (Bruker Biospin GmbH, Rheinstetten, Germany) operating at ^{1}H frequency of 400.3 MHz, ^{13}C frequency of 100.7 MHz and ^{31}P frequency of 162.1 MHz and equipped with a 5 mm BBF/F/H TBO iProbe head and sample case plus an autosampler was used to record the solution NMR spectra. The ^{1}H spectra were recorded applying a 30° pulse averaging 32 scans. The ^{31}P spectra were recorded applying a 30° pulse averaging 512 scans. The ^{13}C spectra were recorded applying a 180° pulse with a 135° read pulse averaging 256 scans.

High pressure liquid chromatography (HPLC) analysis was performed on a PerkinElmer LC300 system equipped with an in-built degasser, a quaternary solvent delivery pump, an autosampler, a column temperature controller, and a photodiode array detector (PDA). SimplicityChrom software was used for system control and data analysis. A Eurospher II 100-5 C18 column (150 x 4 mm) was used for chromatographic separation. The mobile phase was composed of 50 % acetonitrile and 50 % DI water. The total run time for each sample was 20 minutes, no gradient was used.

Chromatograms were recorded at an absorbance of 225 nm. The flow rate was 0.5 mL min^{-1}, the sample injection volume was 5 µL, and the column temperature was set at 25 °C. The sample was diluted by a factor of ten prior to injection.

Electrochemical experiments were carried out in an argon filled UniLab MBraun glove box. Glassware was dried in a heating oven and stored under vacuum for a minimum of 2 h prior to use. Only dried and degassed solvents from a MBraun SPS 7 solvent purifier were used and stored over a 5 Å molecular sieve.

All electrochemical measurements were performed by using a CH Instruments Potentiostat 760 E with corresponding software. As a working electrode a 3 mm Ø glassy carbon electrode (CH Instruments, USA) was used, a platinum wire was used as a counter electrode and an $Ag/AgNO_3$ reference electrode (CH Instruments, USA) containing 1 mM $AgNO_3$ solution in acetonitrile and stored in 0.1 M $[Bu_4N]PF_6$ solution. All cyclic voltammetry measurements were carried out at room temperature in an argon filled glove box and calibrated against the $Fc^{+/0}$ couple. A scan rate of 100 mV/s was used. To obtain the potential-pK_a diagram for the Pourbaix analysis, 1 mM POM was mixed with 4 mM organic acid after taking an initial voltammogram. All measurements were calibrated against the $Fc^{+/0}$ couple after completion of the CV measurements in presence of the organic acids.

Thermogravimetric analysis (TGA) was performed on METTLER TOLEDO TGA 2 STARe system at a heating rate of 10 K min-1 under air flow in a polycrystalline Al2O3 crucible in a temperature range of 30° to 650 ^{o}C.

UV/Vis/NIR spectroscopy was performed on a Cary 3500 UV/Vis/NIR spectrophotometer equipped with a Xenon flash lamp (250 Hz). Measurements were performed in quartz glass cuvettes (d = 10.0 mm).

High resolution electrospray ionization mass spectrometry (ESI-MS) was carried out on an Agilent 6545 QTOF-HRAM-MS system in negative ion mode at a drying gas temperature of T = 180 °C.

Chemicals: All chemical reagents were obtained commercially and used as received unless stated otherwise. $Na_4[(TiOH)PW_{11}O_{38}]$ (**Na_4\{TiOH\}**) was synthesized using a modified literature procedure.[1]

2. Experimental Section

2.1 Synthesis of $Na_4[(TiOH)PW_{11}O_{38}]$ (Na_4\{TiOH\})

Phosphotungstic acid hydrate (1.003 g, 0.347 mmol, 1 eq.) was dissolved in 30 mL distilled water. Sodium carbonate was added until the pH of the solution reaches 5.5. Titanium tetrachloride (0.05 mL, 0.455 mmol, 1.3 eq.) was added to the solution dropwise, the resulting reaction mixture was stirred for 10 minutes at room temperature followed by 20 minutes stirring at 100 °C. After cooling the reaction solution to room temperature any remaining precipitate was removed by centrifugation and the solvent of the supernatant removed under reduced pressure. The raw product was dissolved in 20 mL water followed by addition of 30 mL ethanol resulting in immediate precipitation. The solvent mixture was removed by centrifugation and the solid was washed with 15 mL ethanol three times and dried at 42 °C under reduced pressure for 16 h. A colorless powder was obtained.

Yield: 615 mg (0.217 mmol, 63 % based on phosphorous)

MW: 2834.02 g/mol

FT-IR (cm^{-1}): 1616.36, 1089.38, 1042.45, 949.10, 891.72, 852.09, 793.64, 709.18, 660.05, 591.19, 502.43, 480.56

^{31}P-NMR: -13.86 ppm (Conditions: counter-cation: Na^+, solvent: water)

2.2 ESI-MS Spectrometry

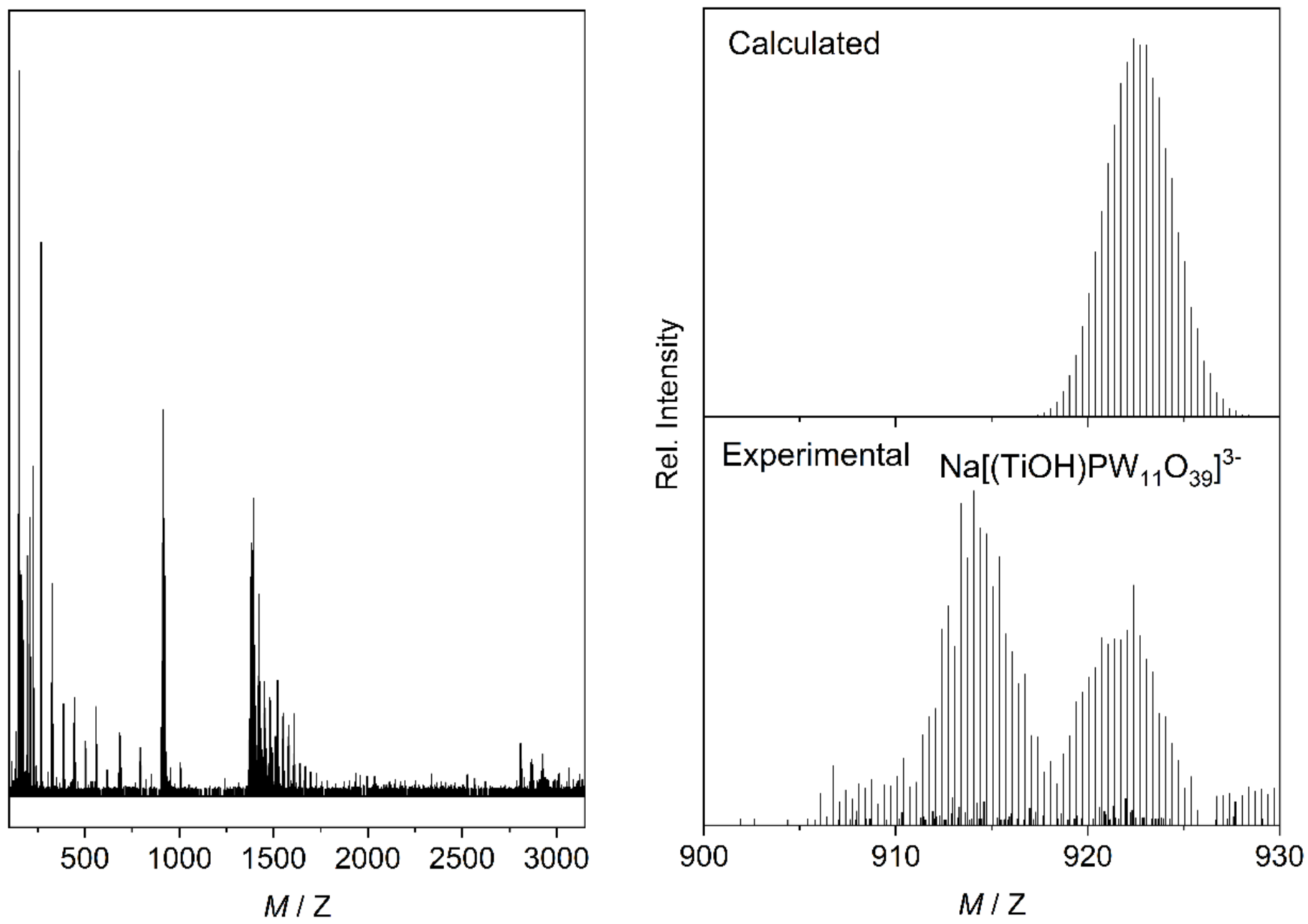

Figure S1: High-resolution negative-ion mode ESI mass spectrum of **Na$_4${TiOH}** (0.05 mM) in H_2O (left), magnified peak and calculated peak (right).

Table S1: Detailed peak assignment of the high-resolution negative-ion mode ESI mass spectrum of **Na$_4${TiOH}**.

Calculated m/z	Observed m/z	Peak assignment
914.051	914.061	$H[(TiOH)PW_{11}O_{39}]^{3-}$
921.378	921.388	$Na[(TiOH)PW_{11}O_{39}]^{3-}$
1371.580	1371.592	$H_2[(TiOH)PW_{11}O_{39}]^{2-}$
1382.577	1382.588	$HNa[(TiOH)PW_{11}O_{39}]^{2-}$
1393.562	1393.576	$Na_2[(TiOH)PW_{11}O_{39}]^{2-}$
2810.148	2810.114	$Na_3[(TiOH)PW_{11}O_{39}]^{-}$

2.3 Benzyl alcohol titration

A 1 mM solution of **Na$_4${TiOH}** was prepared by dissolving 14 mg in 5 mL 0.1 M citrate buffer (pH 5.75). To the aqueous solution was added a solution of 10 – 20000 equivalents (in relation to **Na$_4${TiOH}**) benzyl alcohol in 20 mL toluene. The mixture was stirred for 5 minutes to equilibrate after which the aqueous phase was extracted using a separation funnel and absorption spectra of the aqueous phase were recorded.

2.4 Cation metathesis protocol and synthesis of (*n*Bu$_4$N)$_4${TiOBn}) and (*n*Bu$_4$N)$_4${TiOH}

To a 1 mM solution containing **Na$_4${TiOBn}** or **Na$_4${TiOH}**, 10 equivalents of (nBu$_4$N)Br (as a saturated aqueous solution) were added. The resulting yellow (**(*n*Bu$_4$N)$_4${TiOBn}**) or colorless (**(*n*Bu$_4$N)$_4${TiOH}**) precipitate was removed by centrifugation and washed 3x with water. The product was dried at 42 °C under reduced pressure for 24 h to yield a powder substance.

Yield: > 95 % (based on phosphorus)

MW: **(*n*Bu$_4$N)$_4${TiOH}** 3711.78 g/mol; **(*n*Bu$_4$N)$_4${TiOBn}** 3801.90 g/mol

FT-IR (cm^{-1}): **(nBu$_4$N)$_4${TiOBn}**: 2961.13, 2937.11, 2873.19, 1719.04, 1475.42, 1379.35, 1247.59, 1159.75, 1087.81, 1058.29, 1031.58, 951.81, 880.30, 792.27, 736.05, 676.66, 621.49, 592.85, 515.18, 490.58, 432.25, 410.99; **(nBu$_4$N)$_4${TiOH}**: 2959.71, 2935.39, 2872.42, 1485.72, 1380.73, 1072.76, 959.34, 884.89, 796.78, 732.51, 693.54, 620.83, 593.50, 515.95, 500.44, 470.19, 431.22, 412.61

^{31}P-NMR: **(nBu$_4$N)$_4${TiOBn}**: -13.75 ppm; **(nBu$_4$N)$_4${TiOH}**: -14.35 ppm (Conditions: cation: nBu$_4$N$^+$, solvent: acetonitrile)

2.5 ATR-IR spectroscopy

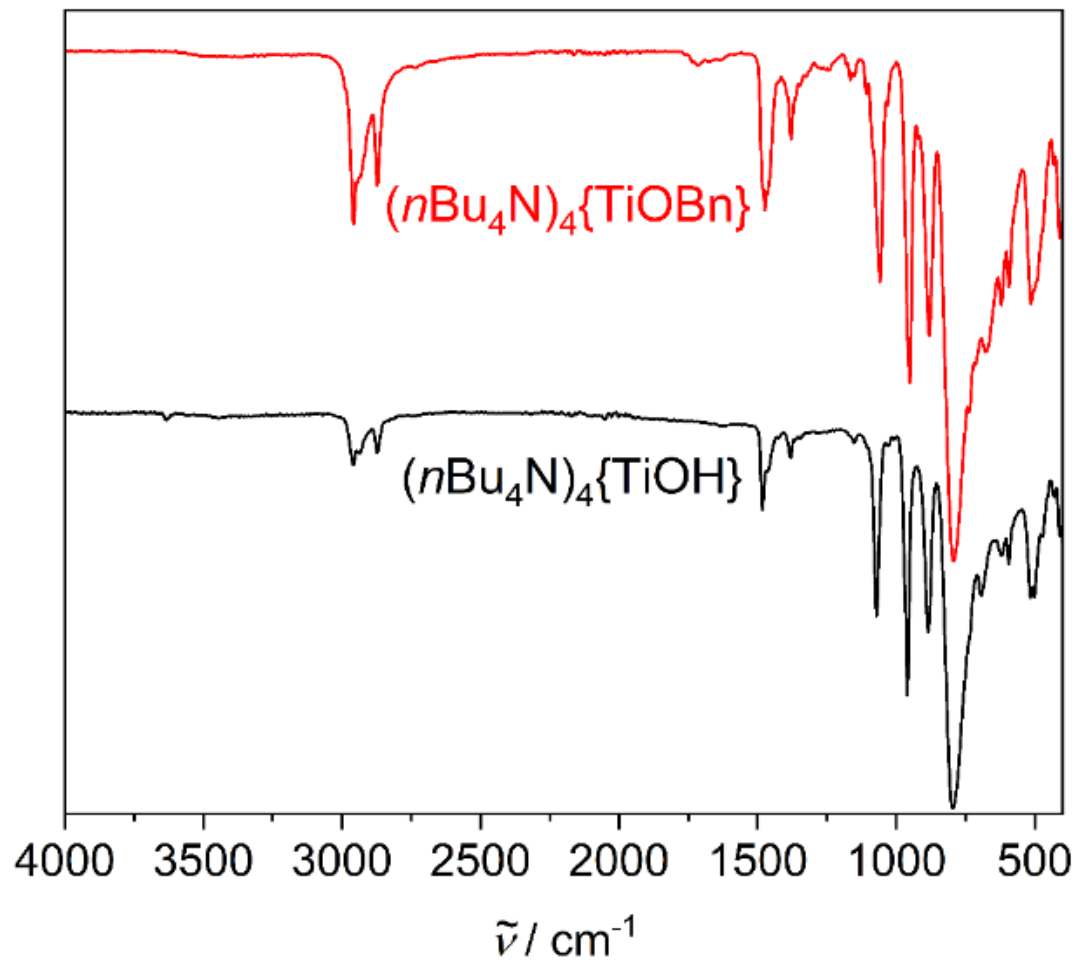

Figure S2: ATR-FT-IR spectra of **(nBu$_4$N)$_4${TiOBn}** (red) and **(nBu$_4$N)$_4${TiOH}** (black).

The ATR-IR spectrum shows prominent bands in the polyoxometalate region (1100 – 700 cm^{-1}) at 1072, 959, 884 and 796 cm^{-1} (ν W=O, W-O-W, vs)[2] which only shift slightly with benzyl alcohol coordination, to 1059, 951, 880 and 792 cm^{-1}. Both compounds show the characteristic C-H vibration bands of the nBu$_4$N$^+$ cation at 2959 and 2873 cm^{-1} (ν -CH$_3$/-CH$_2$-, m) but additionally **(nBu$_4$N)$_4${TiOBn}**) shows bands corresponding to benzyl alcohol at 1719 (ν =CH, vw), 1248, 1159 (ν C=C, vw) and a shoulder at 736 cm^{-1} (ν =CH, vw).[3]

2.6 Thermogravimetric analysis

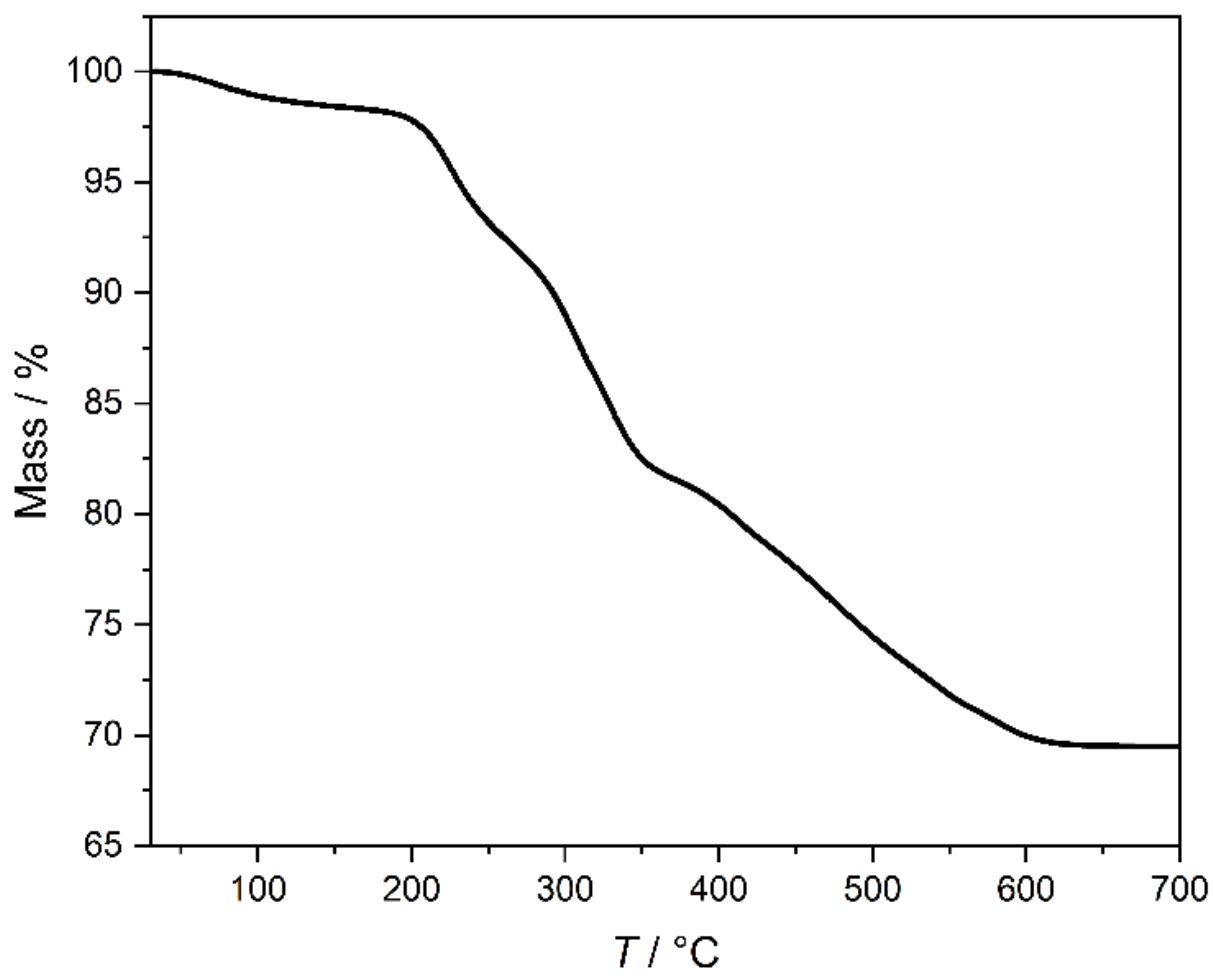

Figure S3: TGA measurement of **(nBu$_4$N)$_4${TiOH}**. The observed weight loss of 27 % between 180 °C and 600 °C indicates the presence of 4 nBu$_4$N$^+$ cations in **(nBu$_4$N)$_4${TiOH}** (calcd.: 26%). Conditions: measurement was performed under air, air flow rate = 60 mL/min, heating rate = 10 °C/min, temperature range = 30 – 600 °C.

2.7 ^{1}H-NMR spectroscopy

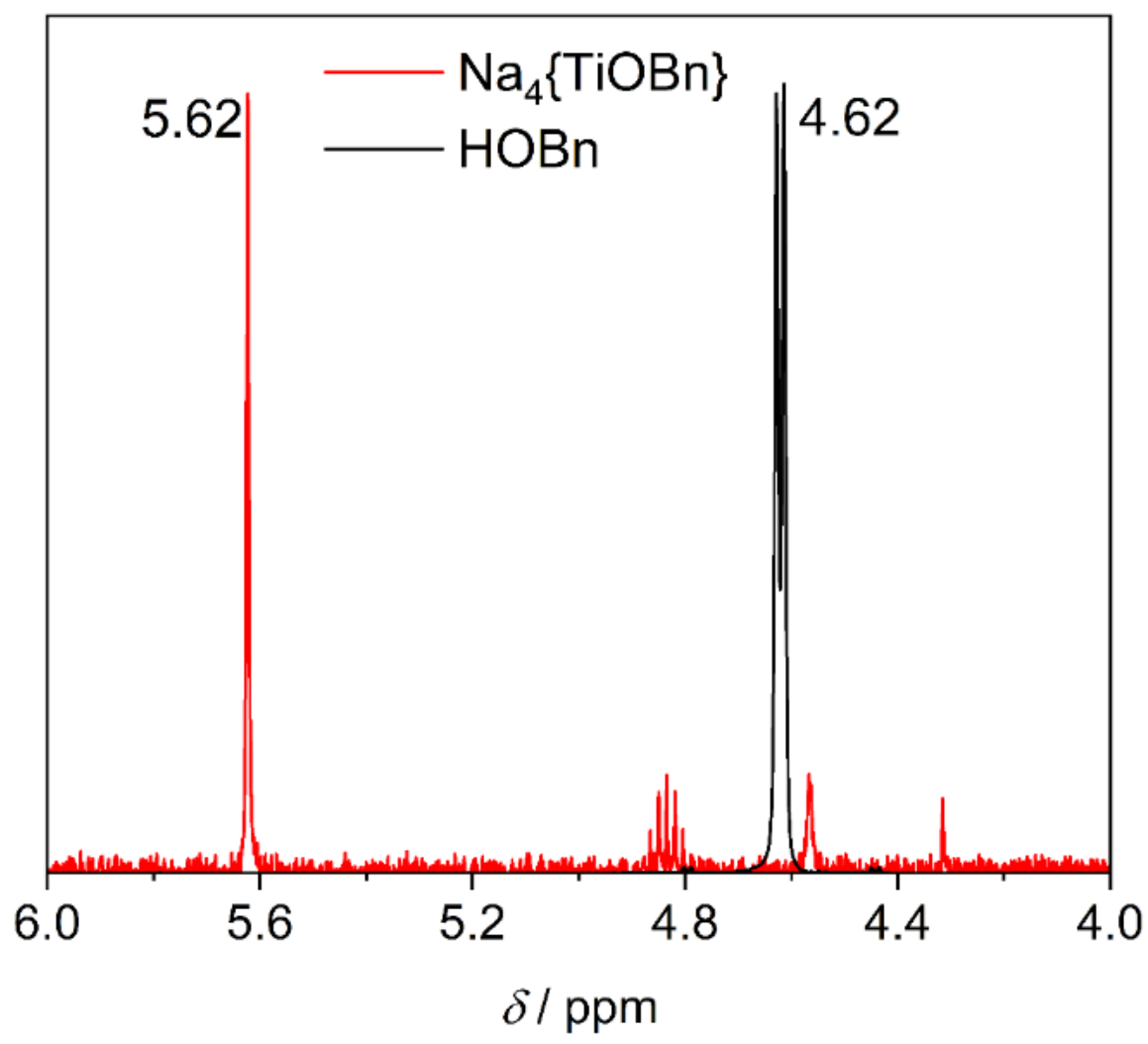

Figure S4: ^{1}H-NMR spectra of **Na_4{TiOBn}** (red) and benzyl alcohol (black), showing the characteristic shift of the benzylic α-CH_2-group of benzyl alcohol upon POM coordination. Solvent: D_2O. In the spectrum of **Na_4{TiOBn}** (red), the signal at 4.59 ppm is residual free BnOH, mMinor unassigned impurity signals are observed at 4.3 ppm and 4.8 ppm; the benzylic OCH_2 of free, non-cluster-coordinated benzyl alcohol is observed at 4.62 ppm (black)..

^{1}H-NMR spectra of benzyl alcohol and **Na_4{TiOBn}** in D_2O were recorded (Figure S4). Benzyl alcohol shows two groups of characteristic ^{1}H-NMR signals, i.e. between 7.3 ppm to 7.5 ppm (aromatic protons of the phenyl ring), and 4.6 ppm (benzylic a-CH_2 group) (Figure S7). For **Na_4{TiOBn}**, the characteristic aromatic proton signals were also observed in the 7.3 ppm to 7.5 ppm region (Figure S8), while a characteristic shift of the benzylic a-CH_2 moiety from 4.6 ppm to 5.6 ppm was observed. This data indicates a polarization of the C-H bond due to coordination to the POM. Similar ^{1}H-NMR upfield shifts have been reported previously for interactions of organic ligands with POM clusters. [4]

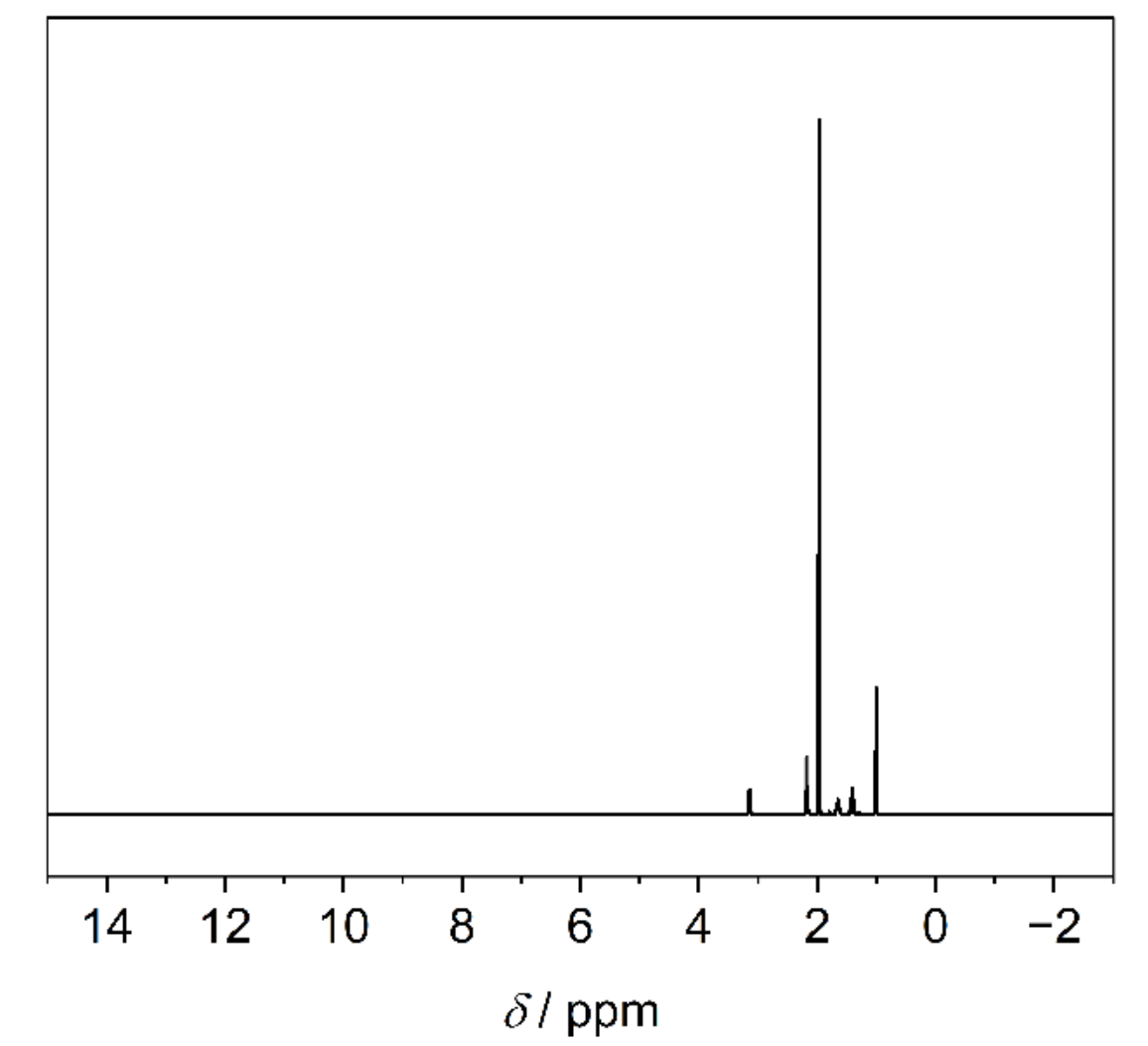

Figure S5: ^{1}H-NMR spectra of **(*n*$Bu_4N)_4${TiOH}**. Solvent: CD_3CN. ^{1}H-NMR (400 MHz, CD_3CN): 3.1 ppm (m, 32H, nBu_4N^+ N-CH_2), 1.60 (m, 32H, nBu_4N^+ CH_2), 1.36 (m, 32H, nBu_4N^+ CH_2), 0.96 ppm (t, 48H, nBu_4N^+ CH_3); residual CHD_2CN at 1.94 ppm, residual H_2O/HDO at 2.13 ppm.

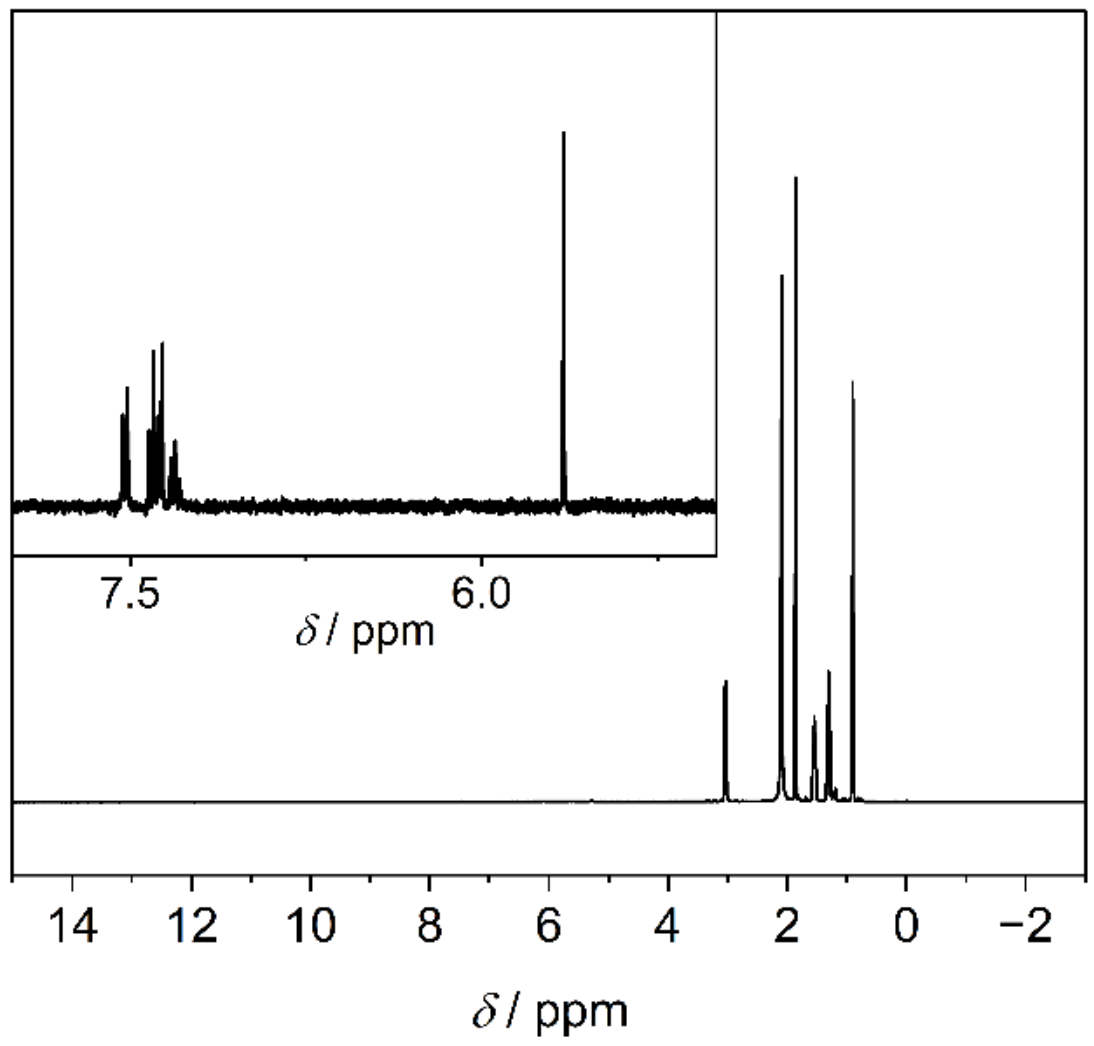

Figure S6: ^{1}H-NMR spectra of **(*n*Bu$_4$N)$_4${TiOBn}**. Solvent: CD_3CN. ^{1}H-NMR (400 MHz, CD_3CN): δ 7.3 ppm to 7.5 pppm (m, 5H, C_6H_5), 5.6 (s, 2H, OCH_2), 3.1 ppm (m, 32H, nBu$_4$N$^+$ N-CH$_2$), 1.60 (m, 32H, nBu$_4$N$^+$ CH$_2$), 1.36 (m, 32H, nBu$_4$N$^+$ CH$_2$), 0.96 ppm (t, 48H, nBu$_4$N$^+$ CH$_3$); residual CHD_2CN at 1.94 and H_2O at 2.13. The benzylic OCH_2 singlet (2H) is weak relative to the 144 nBu$_4$N$^+$ protons per formula unit. Note that integral comparison is not meaningful as there is a BnO$^-$ / OH$^-$ ligand exchange equilibrium in solution. Inset: expansion of the 5 to 8 ppm region.

2.8 ESI Mass Spectrometry

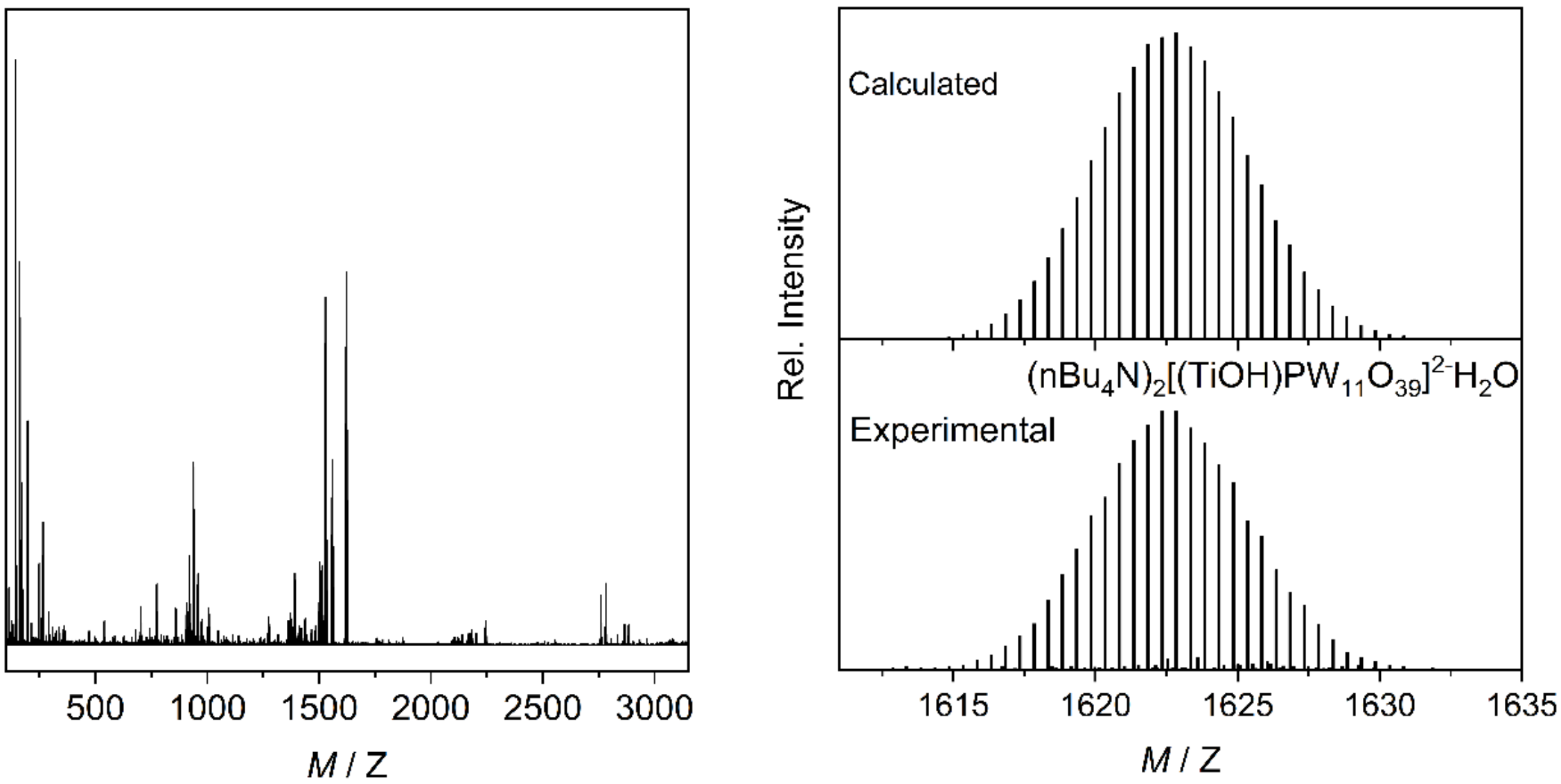

Figure S7: High-resolution negative-ion mode ESI mass spectrum of **(*n*Bu$_4$N)$_4${TiOH}** (0.05 mM) in MeCN (left), magnified peak and calculated peak (right).

Table S2: Detailed peak assignment of the high-resolution negative-ion mode ESI mass spectrum of **(*n*Bu$_4$N)$_4${TiOH}**.

Calculated m/z	Observed m/z	Peak assignment
**689.788**	689.785	[(TiOH)PW$_{11}$O$_{39}$] H$_2$O^{4-}
**920.054**	920.056	H[(TiOH)PW$_{11}$O$_{39}$] H$_2$O^{3-}
**1000.480**	1000.475	(*n*Bu$_4$N)[(TiOH)PW$_{11}$O$_{39}$] H$_2$O^{3-}
**1501.224**	1501.221	(*n*Bu$_4$N)H[(TiOH)PW$_{11}$O$_{39}$] H$_2$O^{2-}
**1622.362**	1622.358	(*n*Bu$_4$N)$_2$[(TiOH)PW$_{11}$O$_{39}$] H$_2$O^{2-}

2.9 ^{1}H-DOSY spectroscopy

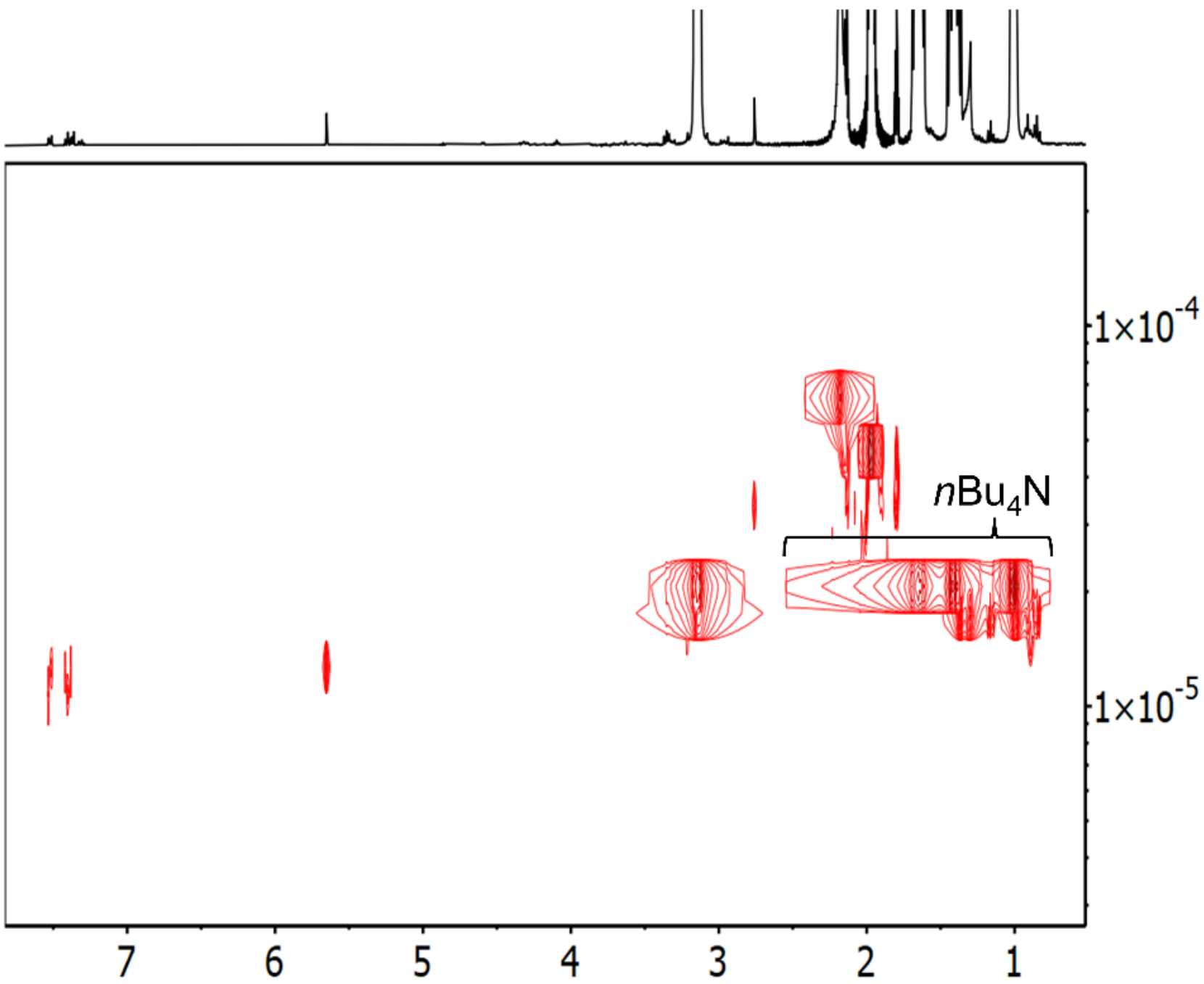

Figure S8: Top: ^{1}H-NMR of **(nBu$_4$N)$_4${TiOBn}**, Bottom: ^{1}H-DOSY of **(nBu$_4$N)$_4${TiOBn}**, the diffusion coefficient of the benzylic α-CH_2-group of the benzyl alcoholate in **(nBu$_4$N)$_4${TiOBn}** is 1.30 * 10^{-5} cm^2/s in CD_3CN. For comparison the diffusion coefficient of the nBu$_4$N$^+$ cations is 1.87 * 10^{-5} cm^2/s in CD_3CN. Solvent: CD_3CN

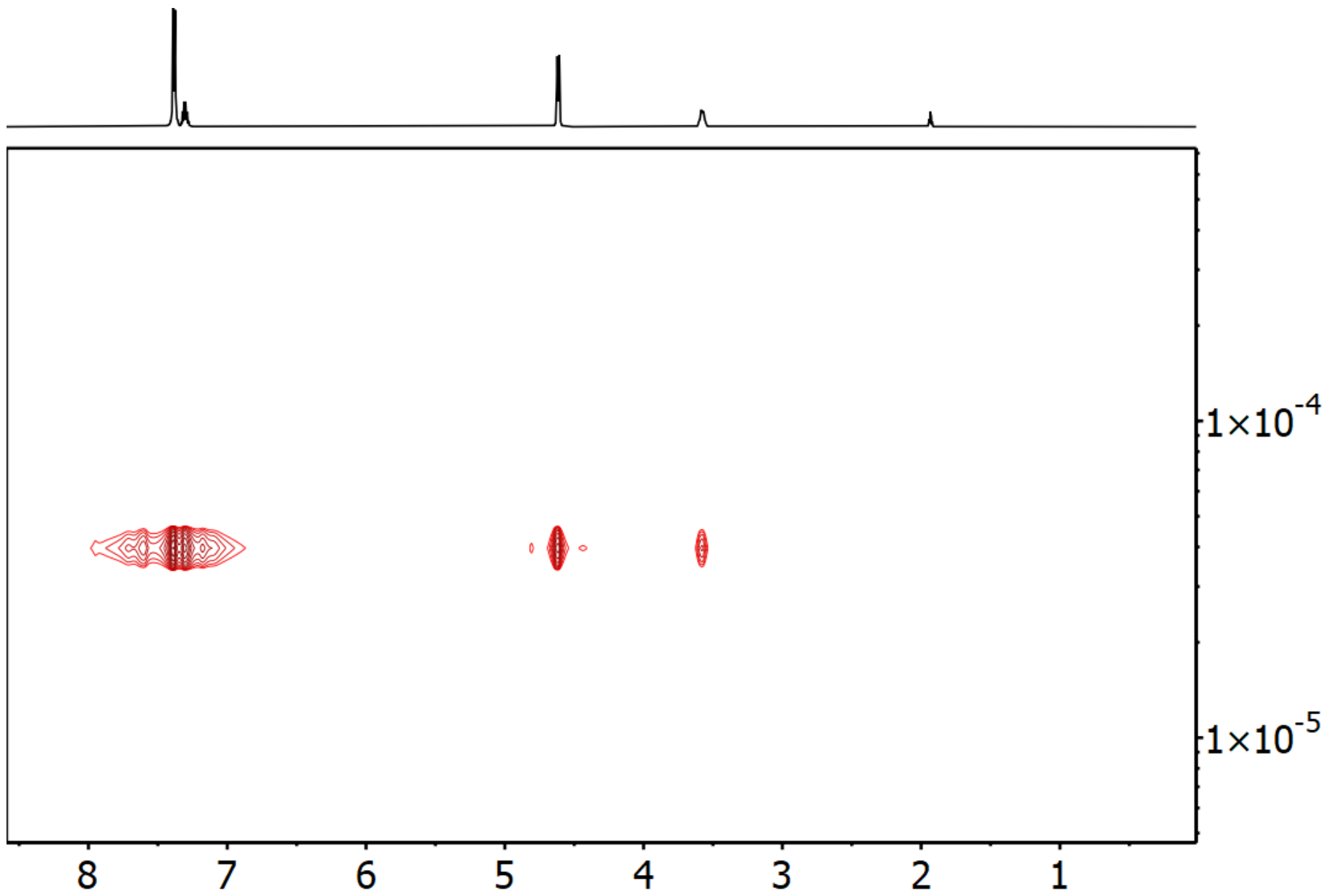

Figure S9: Top: ^{1}H-NMR spectrum of benzyl alcohol. Bottom: ^{1}H-DOSY spectrum of benzyl alcohol, the diffusion coefficient of the benzylic α-CH_2-group of the benzyl alcohol in is 3.94 * 10^{-5} cm^2/s in CD_3CN. ^{1}H-NMR (400 MHz, CD_3CN): δ 7.3 ppm to 7.5 pppm (m, 5H, C_6H_5), 4.8 (s, 2H, OCH_2), 3.6 ppm (m, 1H, OH), residual CHD_2CN at 1.94. Solvent: CD_3CN

^{1}H-DOSY-NMR of **(nBu$_4$N)$_4${TiOBn}** was used to compare the diffusion coefficients of BnOH and **(nBu$_4$N)$_4${TiOBn}** based on monitoring of the α-CH_2 moiety ^{1}H-NMR signal under identical experimental conditions (Figure S6). For the POM, we determined a diffusion coefficient of 1.30 · 10^{-5} cm^2/s, which is in line with previous reports on POM diffusion coefficients in water.[5] In contrast, for BnOH, we observe a diffusion coefficient of 3.94 · 10^{-5} cm^2/s,

thereby allowing the unambiguous differentiation between benzylic groups bound to the POM, or diffusing freely as non-POM-bound BnOH species.

2.10 UV-VIS spectroscopy after benzyl alcohol oxidation

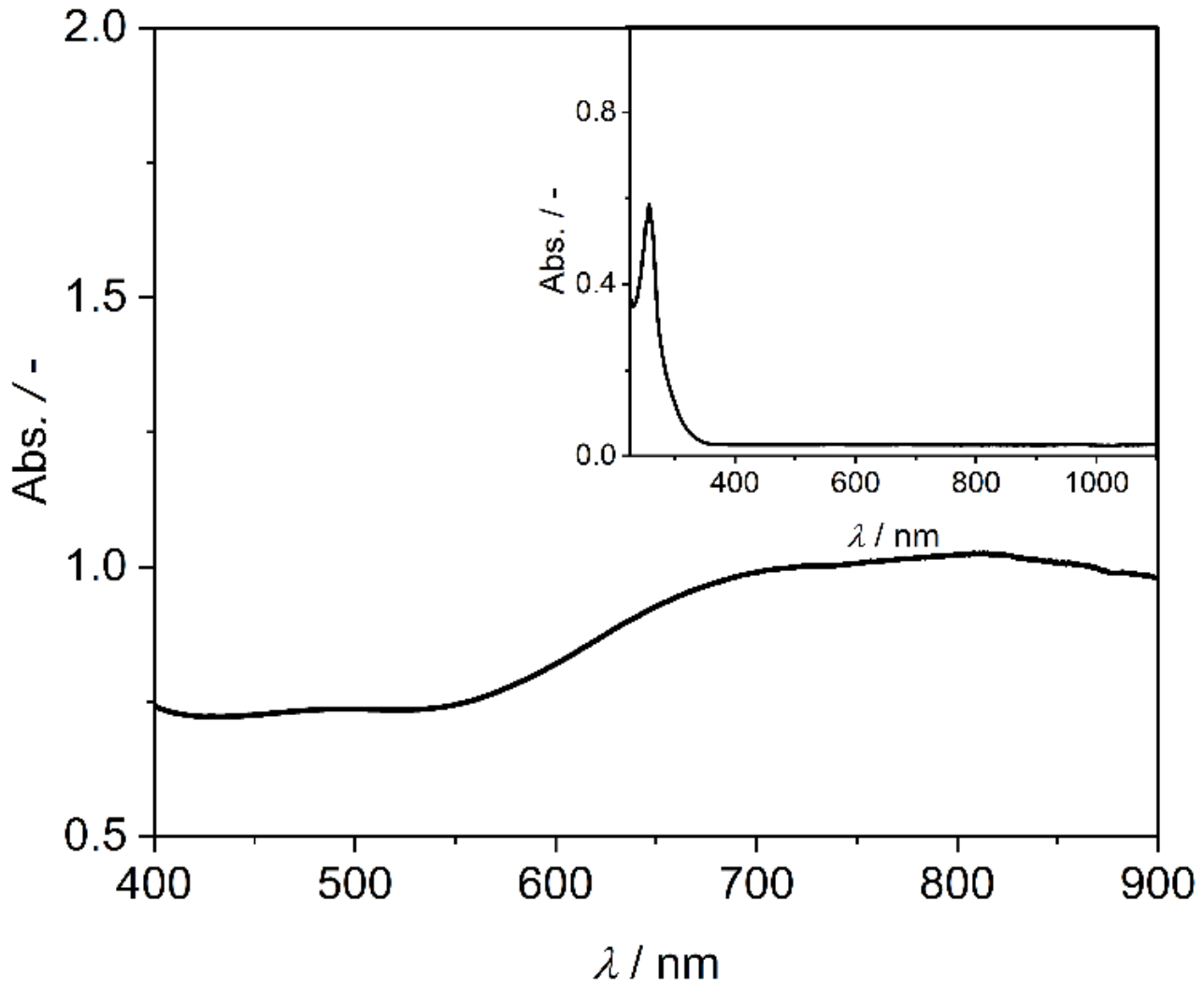

Figure S10: UV-Vis-NIR absorption spectra of the benzyl alcohol oxidation reaction after irradiation ($\lambda_{irradiation}$ = 470 nm, $t_{irradiation}$ = 72 h), showing the formation of the characteristic intervalence charge-transfer (IVCT) assigned to a reduced **{TiOH}** species. Inset: UV-Vis-NIR absorption spectrum of native, non-reduced **Na_4{TiOH}**. Conditions: solvent: aqueous citrate buffer (0.1 M, pH = 5.75), [**{TiOH}**]= 25 mM, [BnOH] = 1 M.

2.11 ^{31}P-NMR spectroscopy

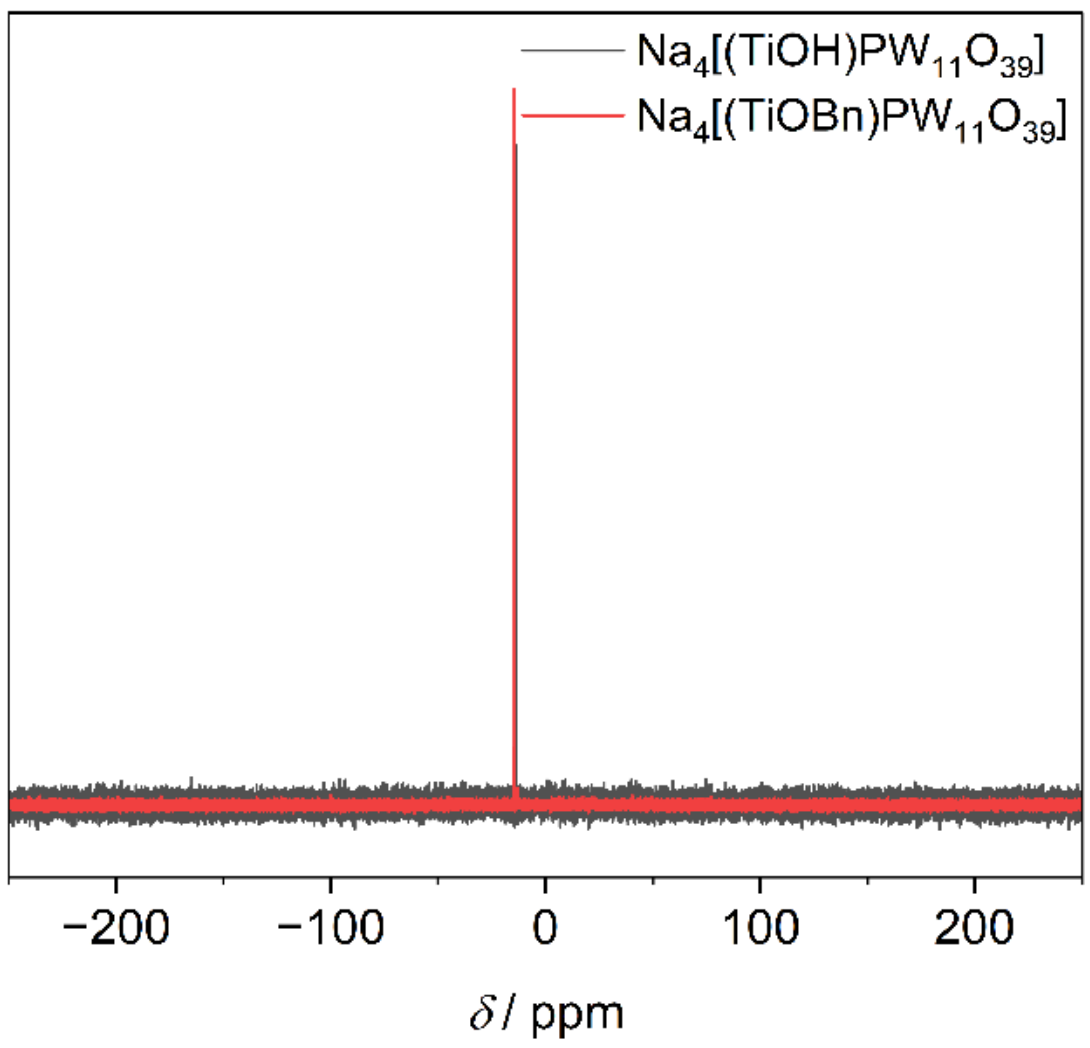

Figure S11: Full width 31P-NMR spectra of **(*n*Bu_4N){TiOH}** (black) and **(*n*Bu_4N){TiOBn}** (red).

2.12 ^{13}C-NMR spectroscopy after benzyl alcohol oxidation

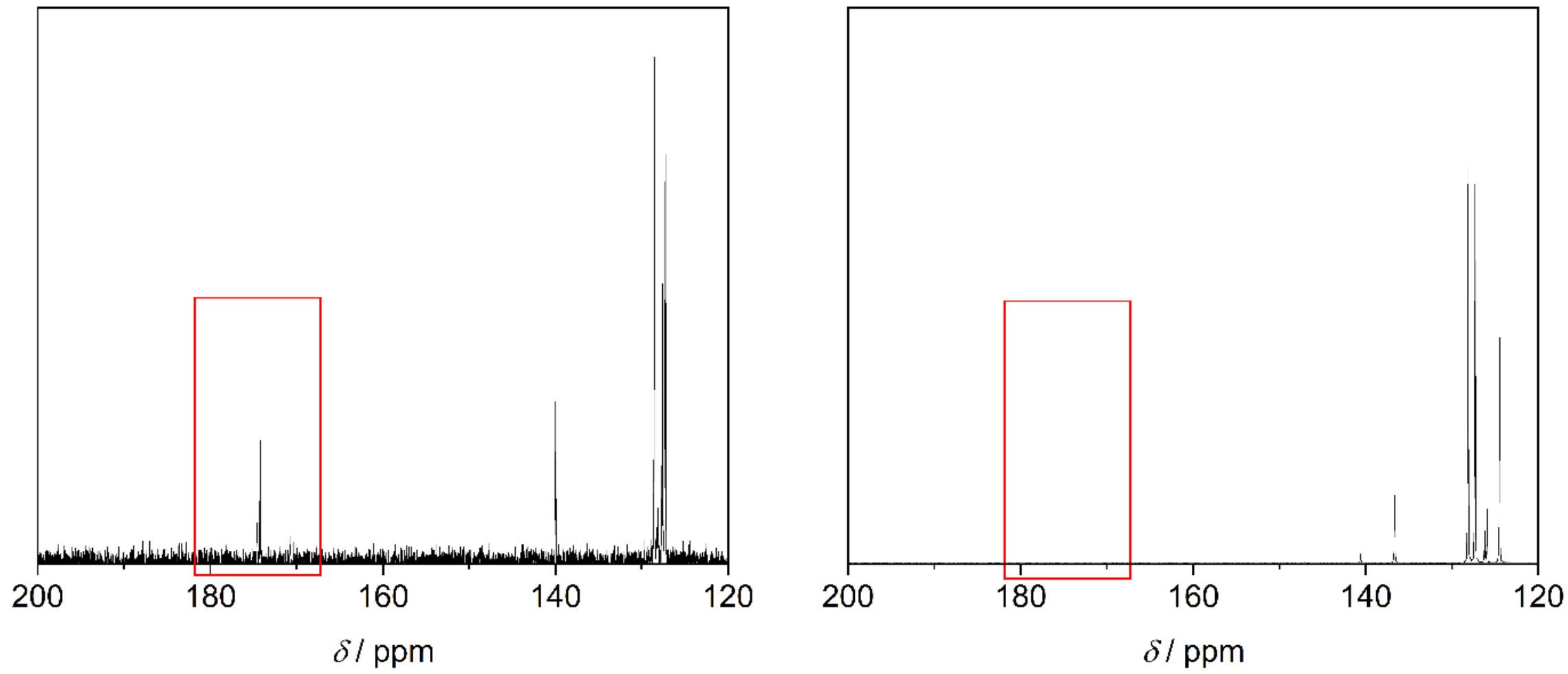

Figure S12: ^{13}C-NMR spectrum of the standard benzyl alcohol oxidation reaction (aqueous phase: left, toluene phase: right) after irradiation ($l_{irradiation}$ = 470 nm, $t_{irradiation}$ = 72 h), the position of the benzoic acid peak is marked in red. No trace of benzoic acid can be detected in the toluene phase, the benzoic acid amounts to 0.015 % of the initial benzyl alcohol concentration resulting most likely from the autooxidation of benzaldehyde in aerated solution. Conditions: solvent: aqueous citrate buffer (0.1 M, pH = 5.75)/toluene, [**{TiOH}**] = 25 mM, [BnOH] = 1 M.

2.13 Cyclovoltammetry of (nBu$_4$N)$_4${TiOBn}

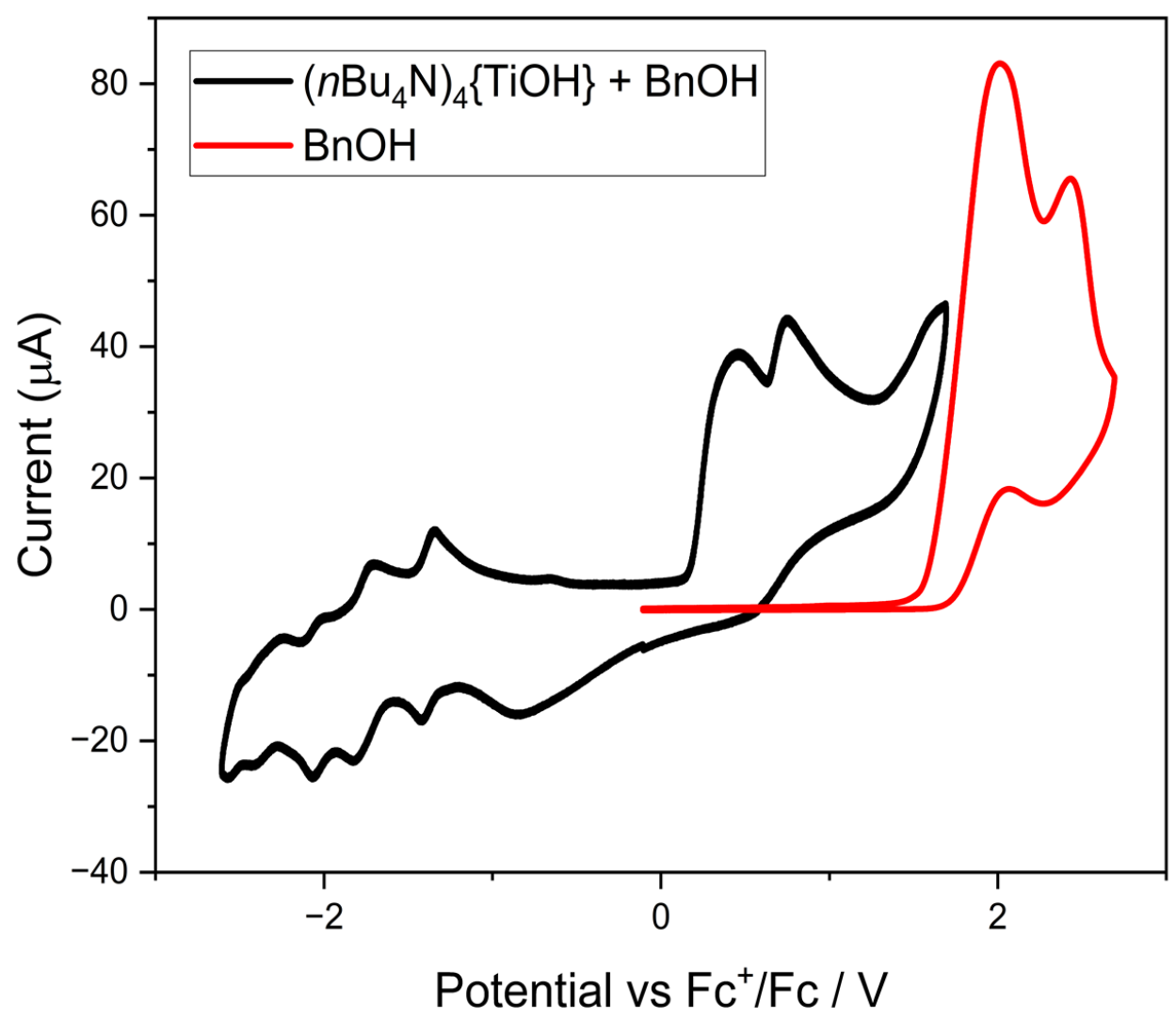

Figure S13: {TiOH}-dependent electrochemical oxidation reactivity of benzyl alcohol: Black: cyclic voltammogram of benzyl alcohol (100 mM) in acetonitrile (containing 0.1 M nBu$_4$NPF$_6$) in the presence of (nBu$_4$N)$_4${TiOH} (1 mM). Red: cyclic voltammogram reference of benzyl alcohol (10 mM) in acetonitrile containing 0.1 mM nBu$_4$NPF$_6$ in the absence of any POM. Conditions: scan rates: 100 mV/s, internal reference: Fc$^+$/Fc.

2.14 Nonaqueous Pourbaix analysis

Porubaix analysis takes advantage of measuring cyclic voltammograms in presence of different reference compounds with certain pK_a values between 5 and 38 (Table S3). The formal redox potentials of each measurement were listed in a so-called potential-pK_a diagram, which shows pK_a-dependent (negative slope) and -independent (horizontal lines) regions for PCET active compounds. For certain reference compounds with pK_a values smaller than the pK_a value of the PCET product POT-H, an anodic (positive) shift of the formal redox potential can be observed (See Figure 5 b)). These pK_a-dependent regions are analogous to pH-dependent regions in aqueous Pourbaix analysis, and their slope shows the ratio of protons and electrons involved in the PCET process.)The general PCET reactions can be described via the following equations:

$[(TiOH)PW_{11}O_{38}(OH)]^{4-} \leftrightharpoons [(TiOH)PW_{11}O_{39}]^{4-} + H^+ + e^-$ [1]

$[(TiOH)PW_{11}O_{36}(OH)_2]^{4-} \leftrightharpoons [(TiOH)PW_{11}O_{39}]^{4-} + 2H^+ + 2e^-$ [2]

$[(TiOBn)PW_{11}O_{37}(OH)_2]^{4-} \leftrightharpoons [(TiOBn)PW_{11}O_{39}]^{4-} + 2H^+ + 2e^-$ [3]

Half wave potentials $E_{1/2}$ of -1.38 V, -1.76 V and -2.21 V vs $Fc^{+/0}$ for **(*n*Bu$_4$N)$_4${TiOH}** and -1.38 V and -1.75 V vs Fc for **(*n*Bu$_4$N)$_4${TiOBn}** were obtained. The pK_a values of **(*n*Bu$_4$N)$_4${TiOH}** and **(*n*Bu$_4$N)$_4${TiOBn}** were calculated from intersection between horizontal lines (formal redox potentials E°) and the linear fits for the pK_a dependent regions from the potential-pK_a diagram. Here we have obtained 23.0 ± 7.6, 25.4 ± 3.8 and 33.5 ± 4.0 for **(*n*Bu$_4$N)$_4${TiOH}** and 16.3 ± 6.4 as well as 22.0 ± 6.3 for **(*n*Bu$_4$N)$_4${TiOBn}** The BDFEs were determined by using the Bordwell equation Eq. 1.[6] Here we were able to assign 52.38 kcal/mol ([3]), 46.90 kcal/mol ([2]) and 47.53 kcal/mol ([1]) for **(*n*Bu$_4$N)$_4${TiOH}** and 43.1 kcal/mol ([5]) and 42.4 kcal/mol ([4]) for **(*n*Bu$_4$N)$_4${TiOBn}**.

The following Table S3 shows all used reference acids for the Pourbaix analysis. Note that due to chemical reactions taking place while adding organic acids significantly fewer data points were collected for **(*n*Bu$_4$N)$_4${TiOBn}** (SI, Figure S12), the applicable acids used for **(*n*Bu$_4$N)$_4${TiOBn}** are marked in blue. Corresponding voltammograms can be found in Figure S11 and Figure S12.

Table S3: pK_a values of reference acids used for non-aqueous Pourbaix-analysis. Note that the pK_a values are taken from Tshepelevitsh and coworkers if not stated other.[7]

**Reference acid**	**Abbreviation**	**p*K*$_a$**
**2,6-Dichloro-anilinium tetrafluoroborate**	Cl_2Anilinium$^+$	5.07
**Diphenylammonium tetrafluoroborate**	$Phe_2NH2\text{-}_2{}^+$	5.98
**2-Chloropyridinium tetrafluoroborate**	2-Cl-PyrH$^+$	6.79
**2-Brompyridium tetrafluoroborate**	2-Br-PyrH$^+$	7.02
**2-Methoxypyridinium tetrafluoroborate**	MeO-PyrH$^+$	9.94
**Anilinium tetrafluoroborate**	Anilinium$^+$	10.64
**N,N-Dimethylanilinium tetrafluoroborate**	DMAH$^+$	11.47
**Pyridinium tetrafluoroborate**	PyrH$^+$	12.53
**2-Methylpyridinium tetrafluoroborate**	2-Me-PyrH$^+$	13.38
**2,6-Lutidiunium tetrafluoroborate**	2,6-LutH$^+$	14.16
**Trimethylphosphonium tetrafluoroborate**	Me_3PH^+	15.48
**Trimethylammonium chloride**	Me_3NH^+	17.61
**1,4-Diaminobutan H**	But-1,4-NH_2	18.31
**Triethylammonium tetrafluoroborate**	Et_3NH^+	18.83
**Dimethylammonium chloride**	$Me_2NH_2^+$	19.03
**Thiophenol**	PhSH	20.91[8,9]
**Benzoic acid**		21.5[10]
**1,1,3,3-Tetramethylguanidinium tetrafluoroborate**	TMGH$^+$	23.35
**1,5-Diazabicyclo[4.3.0]non-5-en H**	DBNH$^+$	23.89
**7-Methyl-1,5,7-triazabicyclo(4.4.0)dec-5-en H**	MTBDH$^+$	25.47
**4-Trifluoromethyl-Phenol**	4-CF_3-Phenol	25.5[11]
**3-Trifluoromethyl-Phenol**	3-CF_3-Phenol	26.5[11]
**Phenol**		29.14
**Indol**		32.57[11]
**Diphenylamin**	Ph_2NH	34.3[9,12]
**Methanol**	MeOH	37.44[9,13]
**Acetonitrile**	MeCN	39.5[9,14]

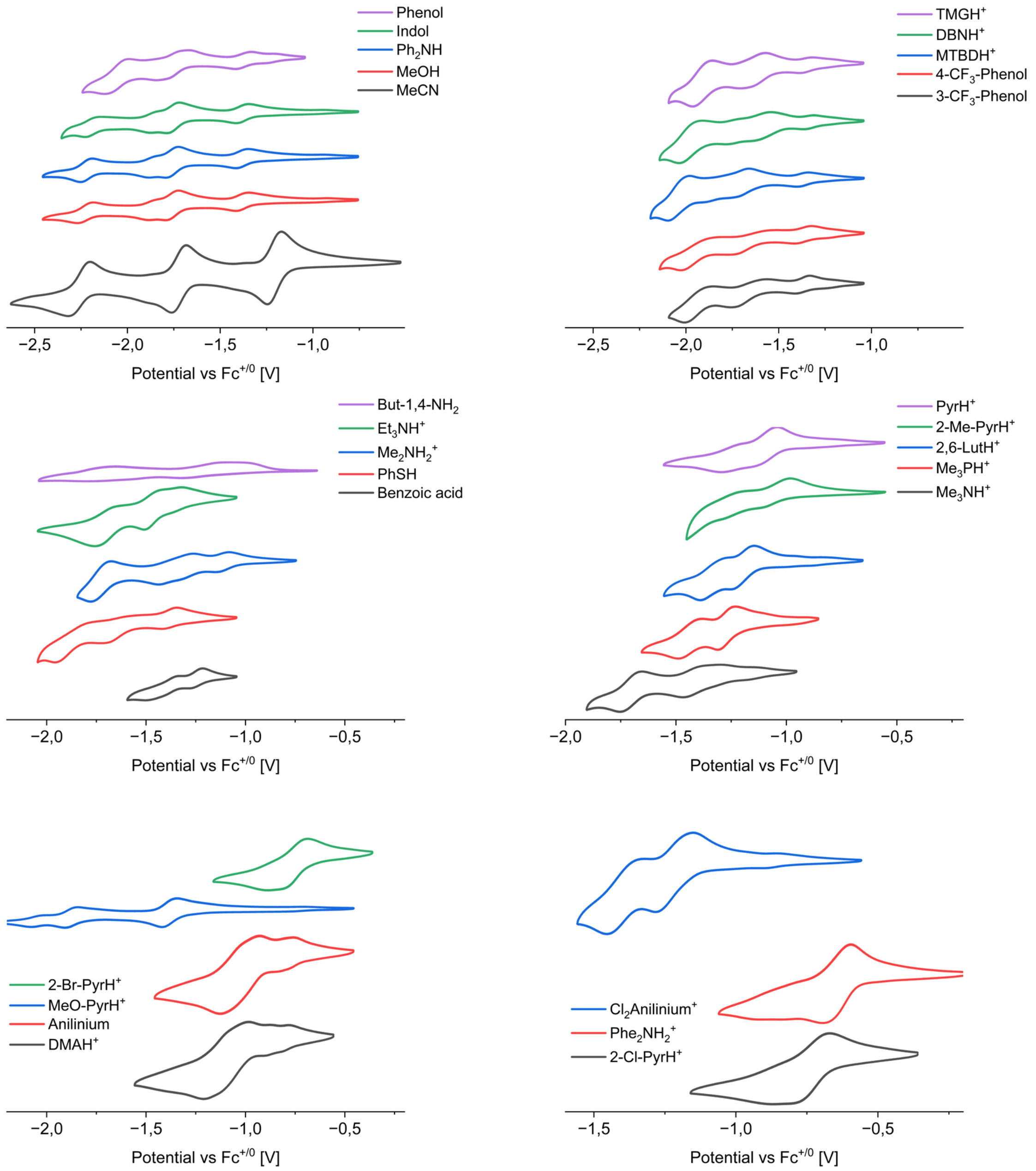

Figure S14: Cyclic voltammograms for Pourbaix-analysis of 1 mM. **(*n*Bu$_4$N)$_4${TiOH}** with 0.1 M (*n*Bu$_4$N)PF_6 supporting electrolyte in acetonitrile in presence of 4 mM of each stated reference acid (Compare Table S3). Each voltammogram was measured at a scan rate of 100 mV/s and referenced versus the $Fc^{+/0}$ couple.

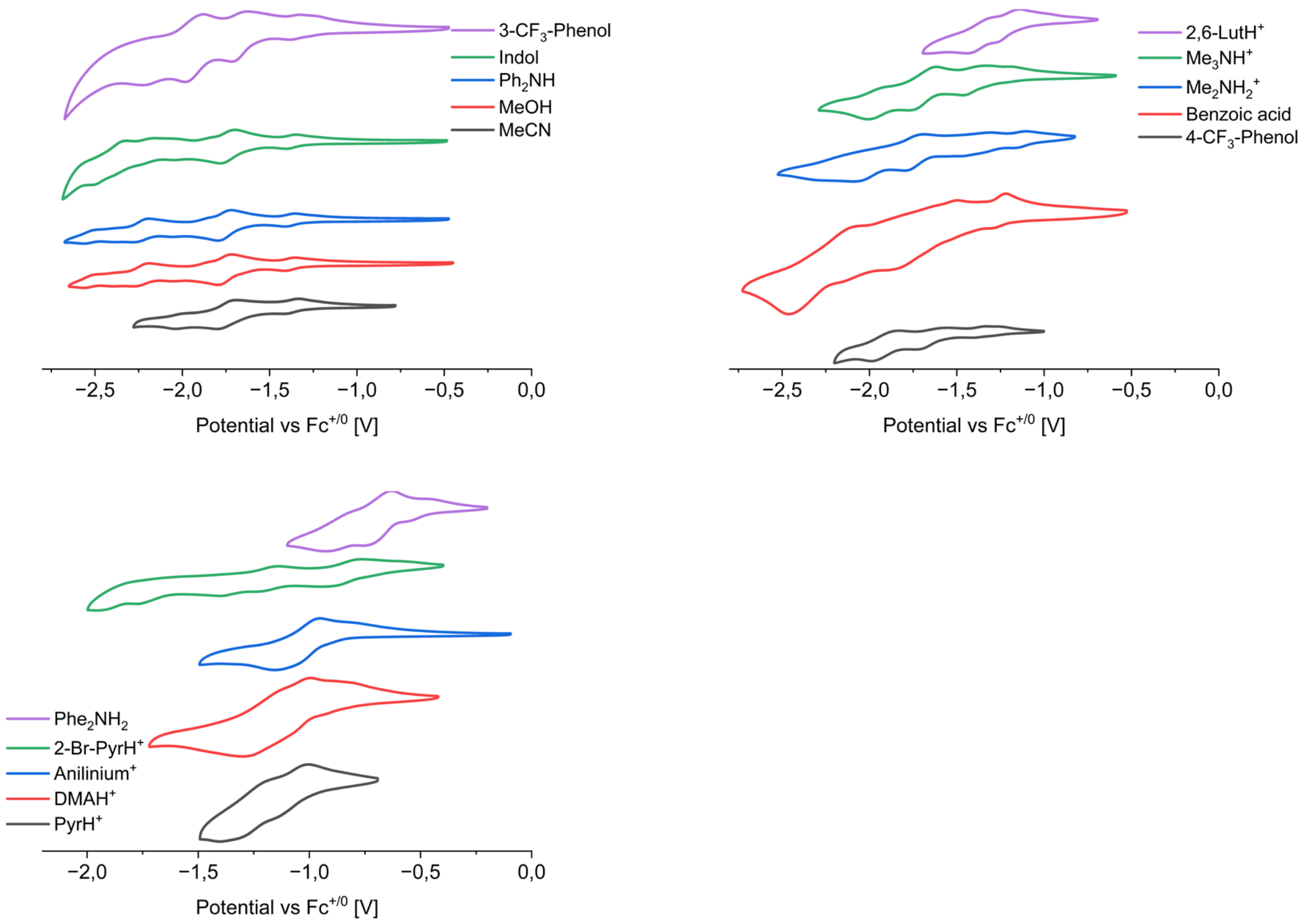

Figure S15: Cyclic voltammograms for Pourbaix-analysis of 1 mM. **(nBu$_4$N)$_4${TiOBn}** with 0.1 M (nBu$_4$N)PF$_6$ supporting electrolyte in acetonitrile in presence of 4 mM of each stated reference acid (Compare Table S3 blue marked acids). Each voltammogram was measured at a scan rate of 100 mV/s and referenced versus the $Fc^{+/0}$ couple.

3. Computational Section

Geometry optimizations and harmonic frequency calculations for all species were performed with the ORCA version 6.1[15] program package at the density functional theory (DFT) level under standard conditions at 298.15 K and 1 atm in conjunction with an implicit solvent model. A conductor-like polarizable continuum model (CPCM, solvent acetonitrile) was used as implemented in ORCA[16] to stabilize the negative charge and to avoid unbound occupied molecular orbitals with positive MO energy eigenvalues in anions. The r^{2}SCAN-3c[17] functional was employed, which makes use of the meta-generalized-gradient approximation (mGGA) and a specifically tailored valence triple-zeta basis set (mTZVPP), [8,9] the D4 London dispersion correction,[18] and a geometrical counterpoise (gCP) correction[19] to account for inter- and intramolecular basis set superposition errors (BSSE). UV-vis spectra were obtained from time-dependent DFT (TD-DFT) calculations within the Tamm-Dancoff approximation (TDA) at the same level of theory, covering up to 2000 roots per species.

Redox potentials were computed using

$$E^{\text{red}}_{\text{abs}} = -\frac{\Delta_r G^{\text{red}}}{n \cdot F}$$

$$E^{\text{red}}_{\text{rel}} = -\left(\frac{\Delta_r G^{\text{red}}}{n \cdot F} + \frac{\Delta_r G^{\text{ox}}(\text{Fc,calc})}{n \cdot F}\right)$$

where n is the number of electrons involved, $F = 96\,485.33\,\text{C}\,\text{mol}^{-1}$ is the Faraday constant; $\Delta_r G^{red}$ is the reaction Gibbs energy of the reduction reaction, and $\Delta_r G^{ox}(Fc,calc)$ is the computed reaction Gibbs energy for oxidation of the ferrocene to the ferrocenium cation.

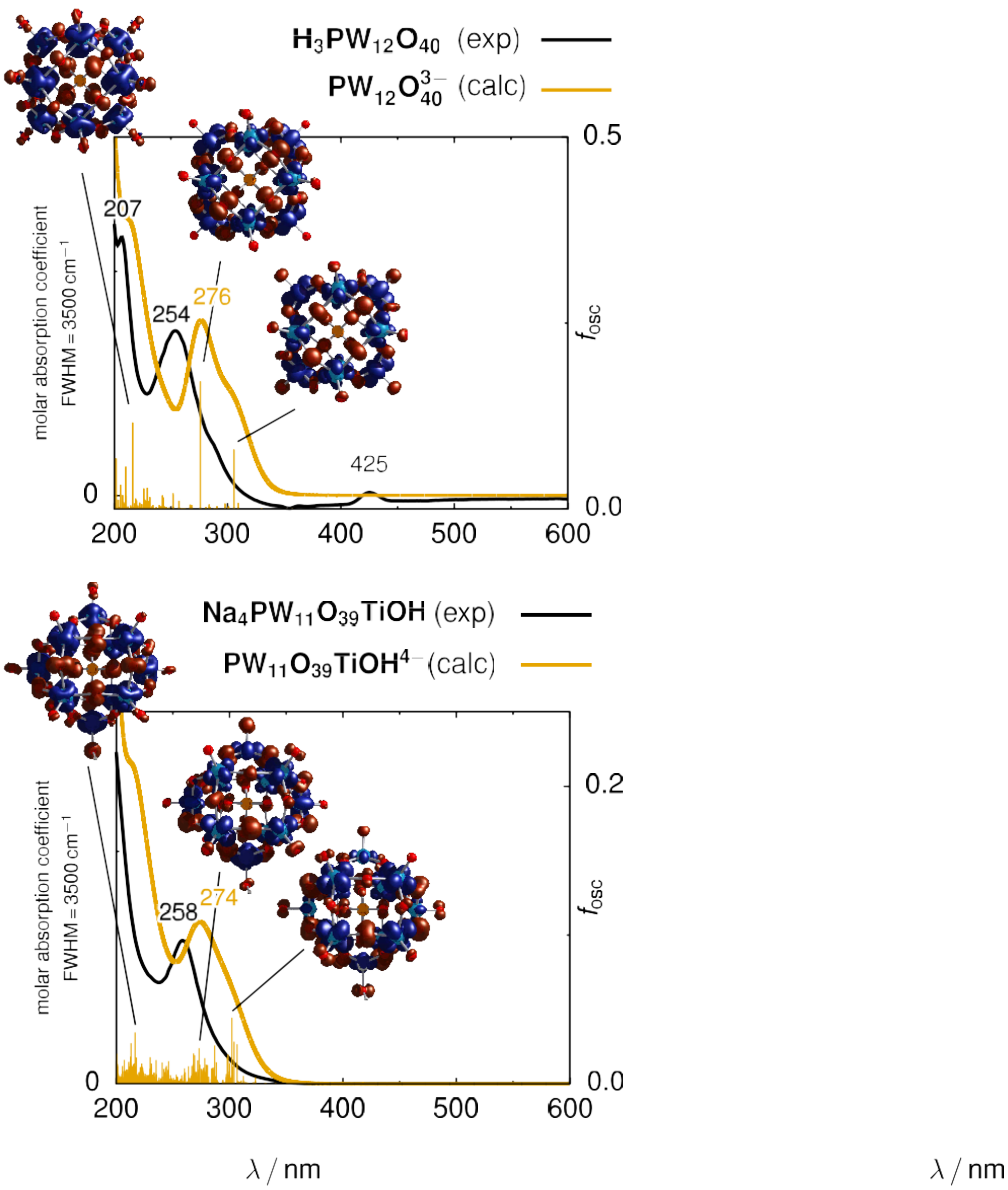

Figure S16: UV-Vis spectrum for the plenary Keggin polyoxotungstate $PW_{12}O_{40}^{3-}$ and for titanium-doped **{TiOH}**, computed at the r^{2}SCAN-3c level. Experimental reference is shown in black color. Difference density plots are shown for selected transitions contributing to the spectra; plot isovalue at 0.001 a_0^{-3} with transitions from red to blue color.

Table S4: Redox reaction enthalpies Δ_rH and Gibbs energies Δ_rG in kcal mol^{-1} for the addition of benzyl alcohol $PhCH_2OH$ to **{TiOH}**, computed at the r^{2}SCAN-3c level.

Reaction							Δ_rH	Δ_rG
^{1}**{TiOH}**$^{4-}$	+	Fc	−→	^{2}**{TiOH}**$^{5-}$	+	Fc$^+$	1.146	1.054
^{2}**{TiOH}**$^{5-}$	+	Fc	−→	^{3}**{TiOH}**$^{6-}$	+	Fc$^+$	1.644	1.581
^{3}**{TiOH}**$^{6-}$	+	Fc	−→	^{2}**{TiOH}**$^{7-}$	+	Fc$^+$	2.191	2.098
^{1}**{TiOBn}**$^{4-}$	+	Fc	−→	^{2}**{TiOBn}**$^{5-}$	+	Fc$^+$	1.165	1.073
^{2}**{TiOBn}**$^{5-}$	+	Fc	−→	^{3}**{TiOBn}**$^{6-}$	+	Fc$^+$	1.658	1.593
^{3}**{TiOBn}**$^{6-}$	+	Fc	−→	^{4}**{TiOBn}**$^{7-}$	+	Fc$^+$	2.212	2.096
		Fc	−→	Fc$^+$			4.619	4.565

Table S5: Enthalpies $\Delta_r H$ and Gibbs energies $\Delta_r G$ in eV for electron affinities of **{TiOH}**$^{n-}$ (relative to **{TiOH}**$^{4-}$) and **{TiOBn}**$^{n-}$ (relative to **{TiOBn}**$^{4-}$), $n = 4\ldots7$, computed at the r^{2}SCAN-3c level. Ionization energies for Fc to Fc$^+$ are also given.

Species	$\langle S^2 \rangle$	$\Delta_r H$	$\Delta_r G$
^{1}**{TiOH}**$^{4-}$	0.000	0.000	0.000
^{3}**{TiOH}**$^{4-}$	2.015	2.799	2.720
^{2}**{TiOH}**$^{5-}$	0.756	−3.473	−3.511
^{4}**{TiOH}**$^{5-}$	3.771	−0.742	−0.821
^{1}**{TiOH}**$^{6-}$	0.000	−6.382	−6.406
^{3}**{TiOH}**$^{6-}$	2.013	−6.447	−6.495
^{2}**{TiOH}**$^{7-}$	1.503	−8.875	−8.963
^{4}**{TiOH}**$^{7-}$	3.769	−8.860	−8.959
^{1}**{TiOBn}**$^{4-}$	0.000	0.000	0.000
^{3}**{TiOBn}**$^{4-}$	2.022	2.503	2.450
^{2}**{TiOBn}**$^{5-}$	0.756	−3.454	−3.492
^{4}**{TiOBn}**$^{5-}$	3.773	−0.611	−0.700
^{1}**{TiOBn}**$^{6-}$	0.000	−6.350	−6.373
^{3}**{TiOBn}**$^{6-}$	2.013	−6.414	−6.464
^{2}**{TiOBn}**$^{7-}$	1.339	−8.832	−8.897
^{4}**{TiOBn}**$^{7-}$	3.769	−8.821	−8.934
Fc	0.000	0.000	0.000
Fc$^+$	0.748	4.619	4.565

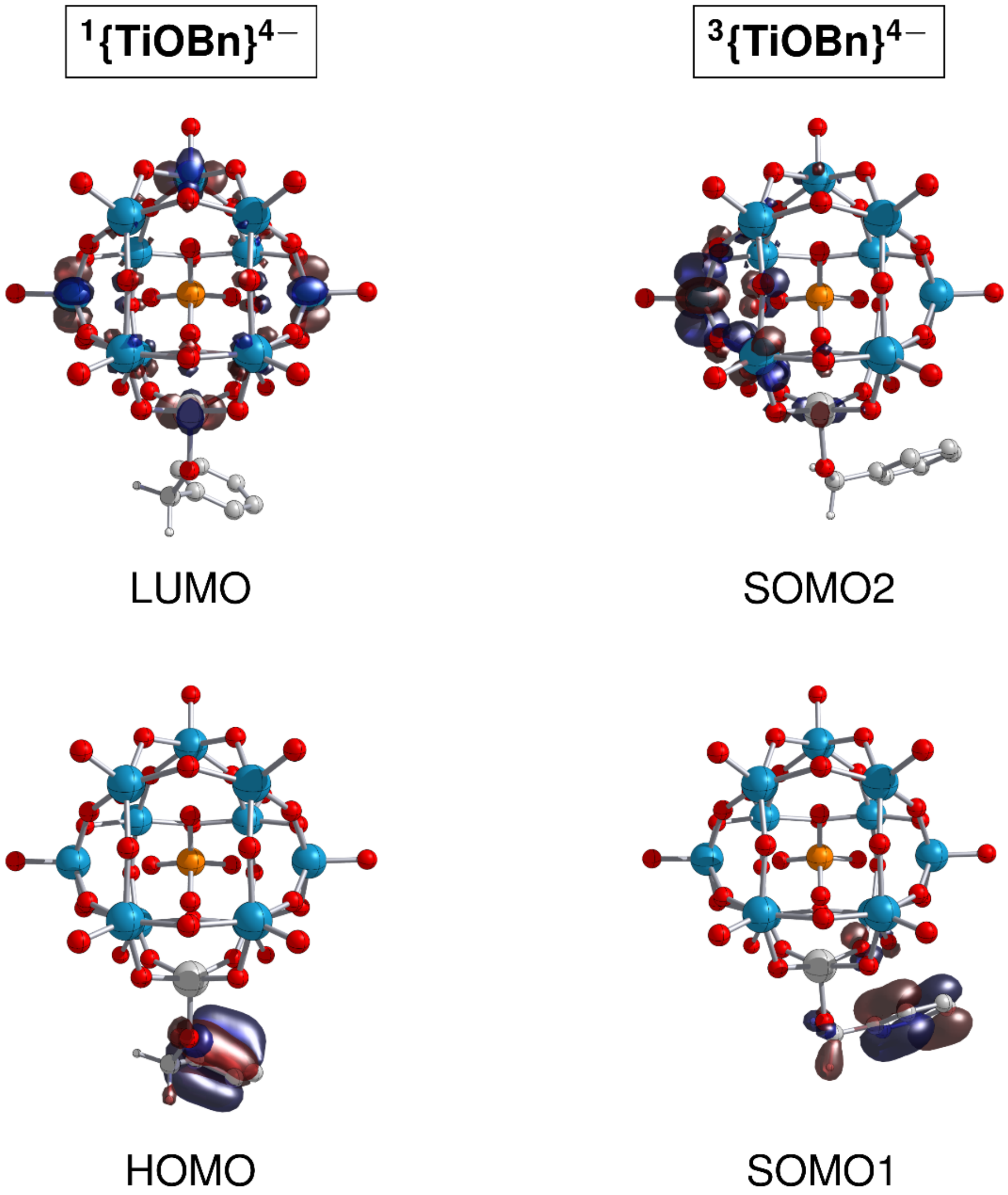

Figure S17: Frontier molecular orbitals for **{TiOBn}**$^{4-}$ in the closed-shell singlet and the triplet electronic states, plotted at an isovalue of +-0.05 $a_0^{\{-3/2\}}$.

4. UV-Vis Transient Absorption Spectroscopy Section

4.1 Experimental setup

A 1 kHz Ti:sapphire oscillator/regenerative amplifier system (Solstice Ace, Spectra Physics) delivering 4 mJ, 35 fs pulses at 800 nm was used for transient UV/Vis pump–probe measurements. Pump pulses at 400 and 267 nm were generated by second and third-harmonic generation of the amplifier output, respectively. Approximately 2 µJ of pump energy, focused to a ~200 µm spot, excited the sample.

The probe beam consisted of a white-light continuum produced by focusing ~1% of the 800 nm beam into a 4-mm sapphire plate. Roughly 30% of this continuum served as a reference, while the remaining 70% was spatially and temporally overlapped with the pump at the sample position. Every second pump pulse was blocked by a synchronized chopper to record pump-induced difference spectra. The pump–probe polarization angle was set to 54.7° to eliminate contributions from rotational relaxation of the photoexcited anisotropic population.

Spectra were collected between 390 and 730 nm using two spectrographs (reference and probe) equipped with 256-pixel linear diode arrays. Pump–probe delays were controlled with a motorized translation stage (Newport DL325). Measurements were performed in a stainless-steel flow cell equipped with two fused silica windows separated by an optical pathlength of 2 mm. A gear pump was used to pump the sample solution with a total volume of about 8 ml. Data acquisition was performed using a LabVIEW interface, and the resulting TA spectra were corrected for temporal shifts caused by group-delay dispersion in the white-light probe. The time resolution of these experiments was about 150 fs.

4.2 UV-Vis transient absorption spectroscopy

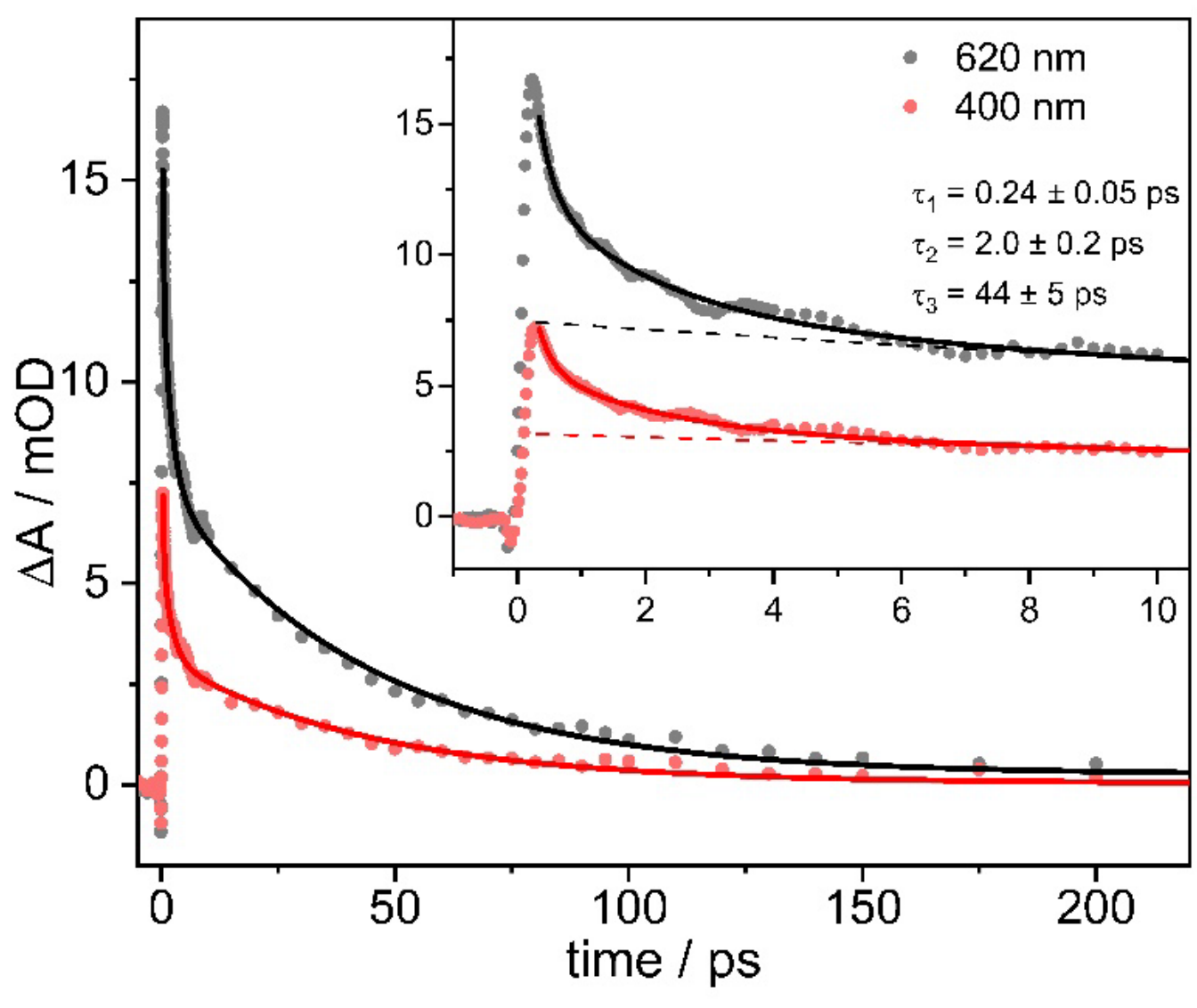

Figure S18: TA time traces for **(nBu$_4$N)$_4${TiOH}** in MeCN after 267 nm excitation. The decays probed at 400 nm (red) and 620 nm (black) were fitted by a sum of three exponential decay functions (full lines), with time constants of τ_1 = 0.24±0.05 ps, τ_2 = 2.0±0.2 ps, and τ_3 = 44±5 ps. The inset zooms into the short time dynamics; the dashed line corresponds to the extrapolated 44 ps decay component.

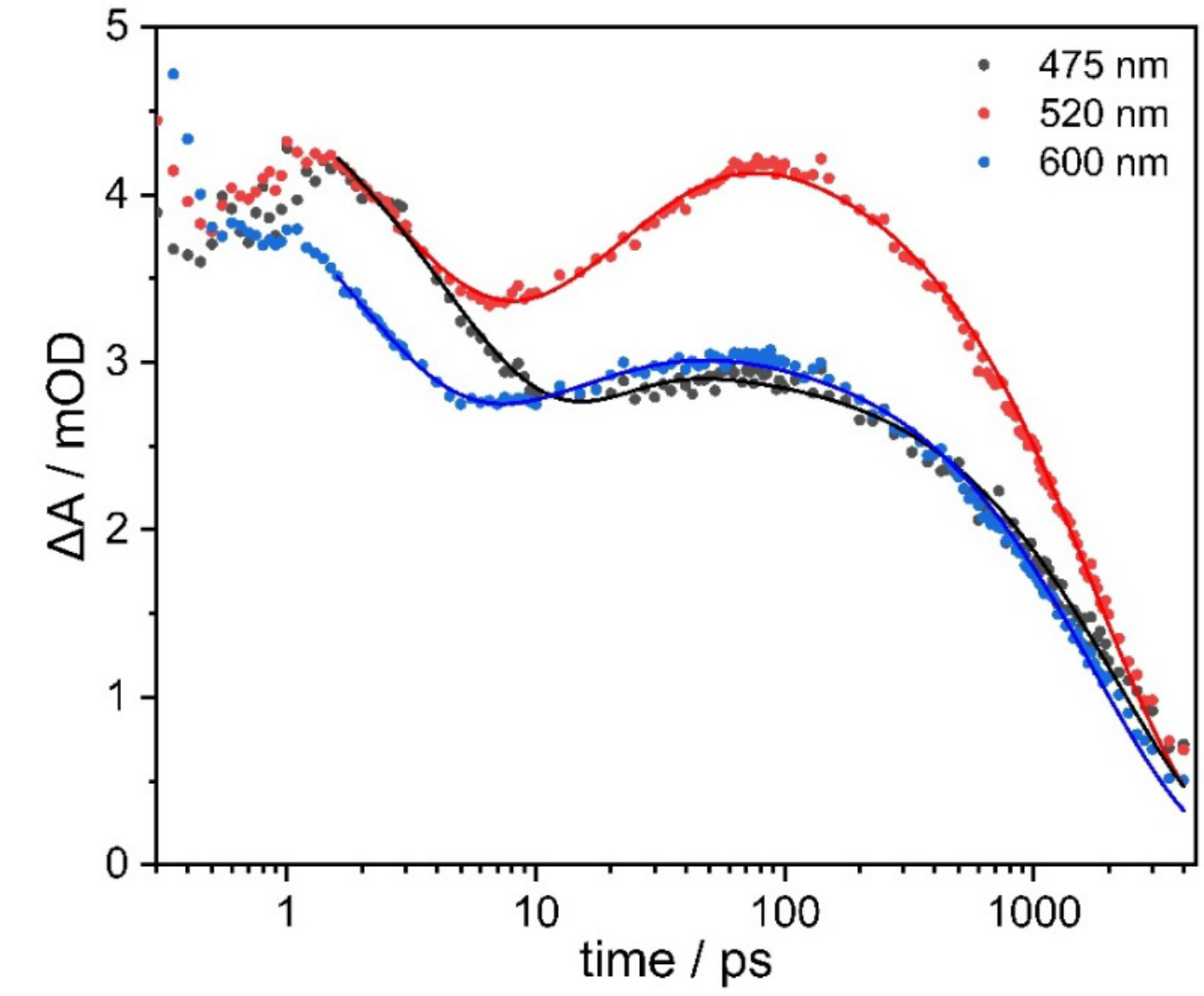

Figure S19: TA time traces for **(nBu$_4$N)$_4${TiOBn}** in acetonitrile after 400 nm excitation. The decays probed at 475 nm (black), 520 nm (red) and 600 nm (blue) were fitted by a sum of three exponential decay functions (full lines), with time constants of τ_1 = 2.5±0.5 ps, τ_2 = 18±4 ps, and τ_3 = 2000±200 ps (note the logarithmic time axis).

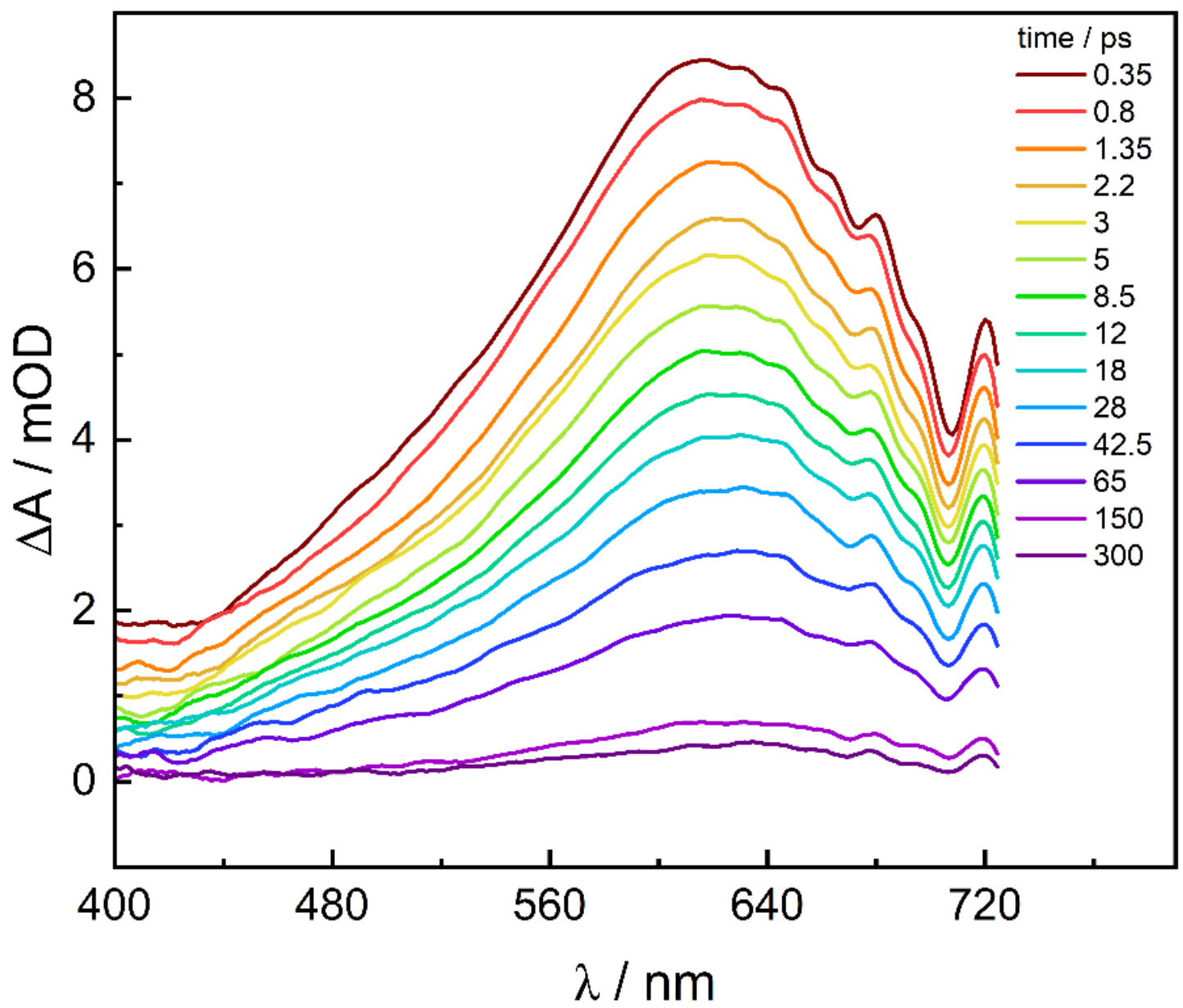

Figure S20: Transient difference spectra of **{PW$_{12}$}** (excitation wavelength 267 nm) at pump-probe delay times as indicated; solvent MeCN, T = 295 K.

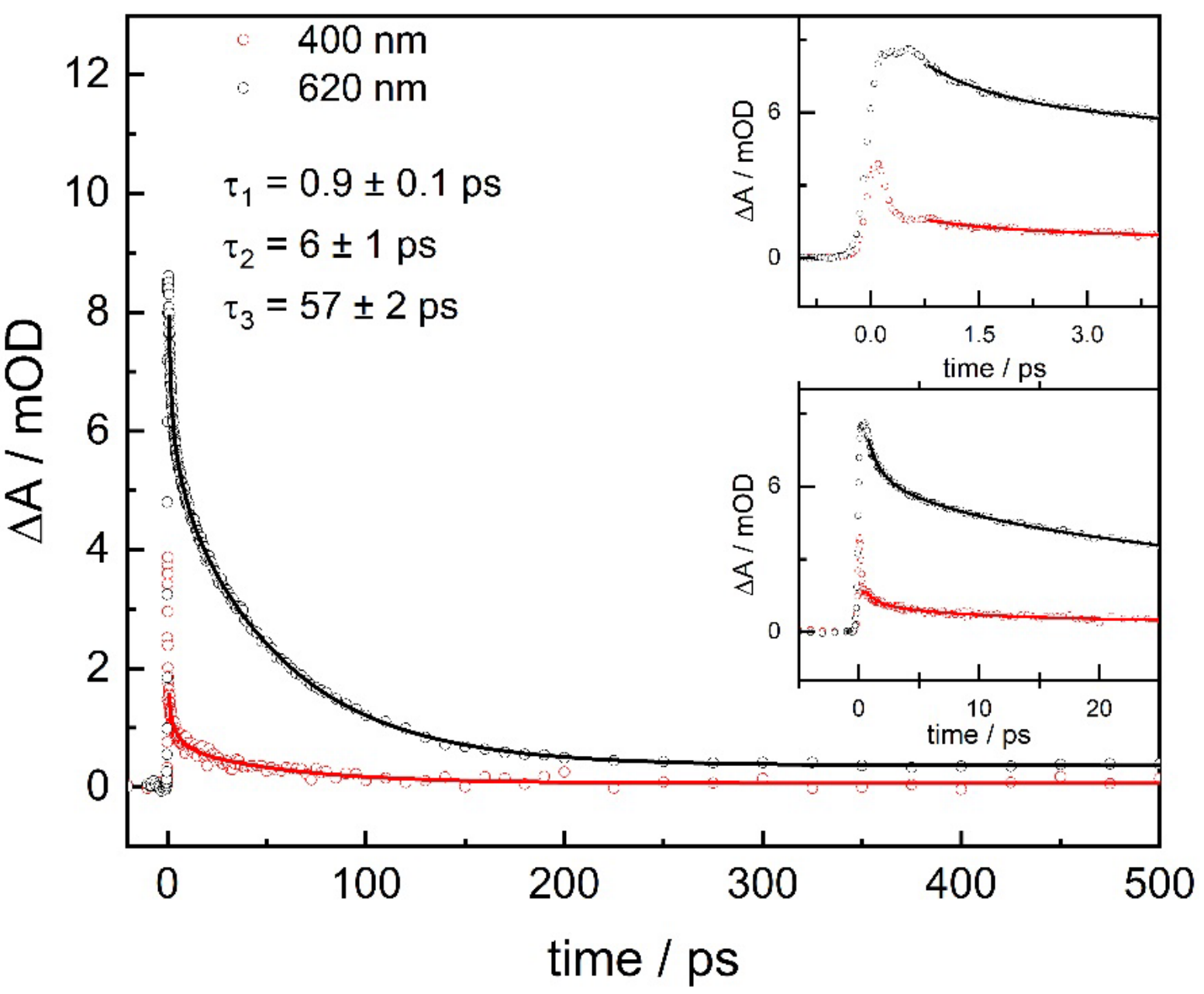

Figure S21: TA time traces for **{PW_{12}}** in MeCN after 267 nm excitation. The decays probed at 400 nm (red) and 620 nm (black) were fitted by a sum of three exponential decay functions (full lines), with time constants of τ_1 = 0.9±0.1 ps, τ_2 = 6±1 ps, and τ_3 = 57±2 ps. The inset zooms into the short time dynamics.

5. References

1 Y. Matsuki, Y. Mouri, Y. Sakai, S. Matsunaga and K. Nomiya, *Eur. J. Inorg. Chem.*, 2013, **2013**, 1754–1761.

2 C. Rocchiccioli-Deltcheff, M. Fournier, R. Franck and R. Thouvenot, *Inorg. Chem.*, 1983, **22**, 207–216.

3 J. National Institute of Advanced Industrial Science and Technolog (AIST), Spectral Database for Organic Compunds SDBS.

4 K. Heussner, K. Peuntinger, N. Rockstroh, L. C. Nye, I. Ivanovic-Burmazovic, S. Rau and C. Streb, *Chemical Communications*, DOI:10.1039/c1cc11859e.

5 F. Leroy, P. Miró, J. M. Poblet, C. Bo and J. B. Ávalos, *Journal of Physical Chemistry B*, 2008, **112**, 8591–8599.

6 F. G. Bordwell, J. Cheng, G. Z. Ji, A. V Satish and X. Zhang, *J. Am. Chem. Soc.*, 1991, **113**, 9790–9795.

7 S. Tshepelevitsh, A. Kütt, M. Lõkov, I. Kaljurand, J. Saame, A. Heering, P. G. Plieger, R. Vianello and I. Leito, *European J. Org. Chem.*, 2019, **2019**, 6735–6748.

8 F. G. Bordwell, *Acc. Chem. Res.*, 1988, **21**, 456–463.

9 H.-Z. Yu, Y.-M. Yang, L. Zhang, Z.-M. Dang and G.-H. Hu, *J. Phys. Chem. A*, 2014, **118**, 606–622.

10 M. Vallaro, G. Ermondi, J. Saame, I. Leito and G. Caron, *Bioorg. Med. Chem.*, 2023, **81**, 117203.

11 A. Kütt, S. Tshepelevitsh, J. Saame, M. Lõkov, I. Kaljurand, S. Selberg and I. Leito, *European J. Org. Chem.*, 2021, **2021**, 1407–1419.

12 F. Maran, D. Celadon, M. G. Severin and E. Vianello, *J. Am. Chem. Soc.*, 1991, **113**, 9320–9329.

13 L. M. Huffman, A. Casitas, M. Font, M. Canta, M. Costas, X. Ribas and S. S. Stahl, *Chemistry–A European Journal*, 2011, **17**, 10643–10650.

14 F. G. Bordwell, J. C. Branca, J. E. Bares and R. Filler, *J. Org. Chem.*, 1988, **53**, 780–782.

15 F. Neese, *Wiley Interdiscip. Rev. Comput. Mol. Sci.*, DOI:10.1002/wcms.70019.

16 M. Garcia-Ratés and F. Neese, *J. Comput. Chem.*, 2019, **40**, 1816–1828.

17 S. Grimme, A. Hansen, S. Ehlert and J. M. Mewes, *Journal of Chemical Physics*, DOI:10.1063/5.0040021.

18 E. Caldeweyher, S. Ehlert, A. Hansen, H. Neugebauer, S. Spicher, C. Bannwarth and S. Grimme, *Journal of Chemical Physics*, DOI:10.1063/1.5090222.

19 H. Kruse and S. Grimme, *Journal of Chemical Physics*, 2012, **136**, 154101.
